# Effect of Electronic Health Record Clinical Decision Support on Contextualization of Care: A Randomized Clinical Trial

**DOI:** 10.1001/jamanetworkopen.2022.38231

**Published:** 2022-10-24

**Authors:** Saul J. Weiner, Alan Schwartz, Frances Weaver, William Galanter, Sarah Olender, Karl Kochendorfer, Amy Binns-Calvey, Ravisha Saini, Sana Iqbal, Monique Diaz, Aaron Michelfelder, Anita Varkey

**Affiliations:** 1 Department of Medicine, College of Medicine, University of Illinois Chicago; 2 Medical Services, Jesse Brown Department of Veterans Affairs (VA) Medical Center, Chicago, Illinois; 3 Center of Innovation for Complex Chronic Healthcare, Edward Hines, Jr VA Hospital, Hines, Illinois; 4 Department of Medical Education, College of Medicine, University of Illinois Chicago; 5 Parkinson School of Health Sciences and Public Health, Loyola University Chicago, Maywood, Illinois; 6 University of Illinois Cancer Center, College of Medicine, University of Illinois Chicago; 7 Department of Family and Community Medicine, College of Medicine, University of Illinois Chicago; 8 Clinical Research Office, Health Sciences Campus, Loyola University Chicago, Maywood, Illinois; 9 Dignity Health, Pacific Central Coast Health Centers, Arroyo Grande, California; 10 Department of Family Medicine, Stritch School of Medicine, Loyola University Chicago, Maywood, Illinois; 11 Loyola University Health System, Chicago, Illinois; 12 Oak Street Health, East Point, Georgia

## Abstract

**Importance:**

Contextualizing care is a process of incorporating information about the life circumstances and behavior of individual patients, termed *contextual factors*, into their plan of care. In 4 steps, clinicians recognize clues (termed *contextual red flags*), clinicians ask about them (probe for context), patients disclose contextual factors, and clinicians adapt care accordingly. The process is associated with a desired outcome resolution of the presenting contextual red flag.

**Objective:**

To determine whether contextualized clinical decision support (CDS) tools in the electronic health record (EHR) improve clinician contextual probing, attention to contextual factors in care planning, and the presentation of contextual red flags.

**Design, Setting, and Participants:**

This randomized clinical trial was performed at the primary care clinics of 2 academic medical centers with different EHR systems. Participants were adults 18 years or older consenting to audio record their visits and their physicians between September 6, 2018, and March 4, 2021. Patients were randomized to an intervention or a control group. Analyses were performed on an intention-to-treat basis.

**Interventions:**

Patients completed a previsit questionnaire that elicited contextual red flags and factors and appeared in the clinician’s note template in a contextual care box. The EHR also culled red flags from the medical record, included them in the contextual care box, used passive and interruptive alerts, and proposed relevant orders.

**Main Outcomes and Measures:**

Proportion of contextual red flags noted at the index visit that resolved 6 months later (primary outcome), proportion of red flags probed (secondary outcome), and proportion of contextual factors addressed in the care plan by clinicians (secondary outcome), adjusted for study site and for multiple red flags and factors within a visit.

**Results:**

Four hundred fifty-two patients (291 women [65.1%]; mean [SD] age, 55.6 [15.1] years) completed encounters with 39 clinicians (23 women [59.0%]). Contextual red flags were not more likely to resolve in the intervention vs control group (adjusted odds ratio [aOR], 0.96 [95% CI, 0.57-1.63]). However, the intervention increased both contextual probing (aOR, 2.12 [95% CI, 1.14-3.93]) and contextualization of the care plan (aOR, 2.67 [95% CI, 1.32-5.41]), controlling for whether a factor was identified by probing or otherwise. Across study groups, contextualized care plans were more likely than noncontextualized plans to result in improvement in the presenting red flag (aOR, 2.13 [95% CI, 1.38-3.28]).

**Conclusions and Relevance:**

This randomized clinical trial found that contextualized CDS did not improve patients’ outcomes but did increase contextualization of their care, suggesting that use of this technology could ultimately help improve outcomes.

**Trial Registration:**

ClinicalTrials.gov Identifier: NCT03244033

## Key Points

**Question:**

Can customized clinical decision support (CDS) tools that provide clinicians with information about patient life challenges improve patient outcomes by helping clinicians contextualize their care?

**Findings:**

In this randomized clinical trial that included 452 audio-recorded patient encounters, customized CDS tools did not improve patients’ outcomes but significantly improved contextualization of their care.

**Meaning:**

This study found that CDS improved contextualization of care, suggesting that the technology has the potential to improve patient outcomes.

## Teaser

This randomized clinical trial assesses the use of clinical decision support tools to provide primary care clinicians with information about contextual factors regarding patient life challenges at the point of care.

## Introduction

Contextualizing care is the process of eliciting and incorporating relevant information about the life circumstances and behavior of individual patients, termed *contextual factors*, into their plan of care.^1^ The process typically includes 4 steps: First, recognizing clues of unaddressed contextual factors (eg, loss of control of diabetes), termed *contextual red flags*; second, asking patients about them, termed *contextual probing*; third, eliciting relevant contextual factors (eg, deteriorating vision in a patient self-administering insulin); and fourth, addressing these factors in the care plan (eg, prescribing prefilled syringes) to contextualize care.^2,3,4^

Contextualizing care is clinically important because it is associated with partial or complete resolution of the presenting contextual red flag, a meaningful health care outcome.^5^ Loss of control of a chronic condition, missed appointments, and frequent emergency department visits are all examples of contextual red flags that improve when their underlying contextual factors are addressed.^6^

Although not all patient presentations contain contextual factors, approximately 40% to 50% do.^6^ Clinicians address them 60% to 70% of the time, leaving substantial room for improvement.^6,7^ These failures to contextualize care are termed *contextual errors*.^7,8,9^

Efforts to improve contextualization of care through audit and feedback have achieved measurable but limited success. In 1 large study,^5^ 666 clinicians received recurring feedback on their performance at contextualizing care during 2 years based on content coding for contextualization of care (4C) analysis of nearly 4500 audio-recorded visits. Attention to patient contextual factors increased from 67% to 72%, with a greater likelihood of improved outcomes compared with a control group, resulting in an estimated cost savings of $25.2 million from avoided hospitalizations. Although the intervention was effective, 28% of contextual factors remained unaddressed.^5^

We hypothesized that customized clinical decision support (CDS) would offer a scalable opportunity to help clinicians contextualize care and, in so doing, would improve patient outcomes. In this study, we tested those hypotheses using CDS designed to provide clinicians information about contextual factors at the point of care.

## Methods

### Study Design and Participants

In this 27-month randomized multicenter clinical trial, primary care attending clinicians were recruited at 2 academic health centers in Chicago using 2 different EHRs, developed by Cerner Corporation (site 1) and Epic Systems (site 2). Physicians were informed that the purpose of the project was to assess whether enhanced clinical decision support that provides information about patient contextual factors could improve clinical decision-making and health care outcomes. They were informed that if they participated, a small number of patients would audio record their visits. The trial protocol (available in Supplement 1) was approved by the Institutional Review Boards at the University of Chicago and Loyola University Chicago, and both clinicians and patients provided written informed consent. This study followed the Consolidated Standards of Reporting Trials (CONSORT) reporting guideline.

All English-speaking adult patients of participating clinicians who could be contacted in advance of their appointments were eligible to participate. Patients were informed that if they participated, they would complete a questionnaire about challenges that might impact their care. They could access it directly in the patient portal or complete it with assistance from a research assistant. When they arrived for their appointment, they received a digital audio recorder to carry into their visit. They were told that it was preferable to conceal the recorder, but that they could reveal it if they felt more comfortable doing so. They were informed that a member of the research team would access their medical record first to note contextual red flags and factors self-identified by patients on the questionnaire, and then several months later to see if key health care indicators noted at the visit had improved. Finally, they were told that their clinician might or might not receive the information they provided, based on random assignment. Patients received a $20 recruitment incentive.

### Intervention

The intervention design, which followed the Five Rights of CDS framework, consisted of 2 elements: a contextual care box (CCB) that appeared in the clinician’s note at the start of the visit notifying them of contextual factors based on patient questionnaire responses and selected passive and active interruptive alerts intended to direct them toward a contextualized care plan and away from a contextual error.^10^ Both were populated by 2 previsit sources of data: the questionnaire and a set of algorithms that extract contextual factors from the patient’s medical record.

The questionnaire included 7 questions designed in a prior study^11^ to elicit a broad range of contextual red flags pertaining to medication, appointment, laboratory testing, and test adherence; declining recommended treatments, tests, and procedures; repeated visits to the emergency department; difficulty accessing equipment or supplies; challenges performing activities important to staying healthy, and other challenges related to self-managing care (eAppendix 1 in Supplement 2). An affirmative response to any item prompted the respondent to select 1 or more contextual factors if present.

In addition, the following contextual red flags were extracted from the patient’s medical record based on rules programmed into the EHR and activated for intervention patients: missed appointments, missed tests and procedures, multiple emergency department visits, loss of control of either diabetes or hypertension while receiving medication, and self-pay status. Each was determined by a set of parameters, such as number of missed appointments in a specific time frame (examples in Table 1). For participants randomized to the intervention, contextual red flags and factors appeared in the CCB (example in eAppendix 2 in Supplement 2). Interruptive alerts were used either to initiate nonmedical interventions that were likely to be beneficial (example in eAppendix 3 in Supplement 2) or to redirect an apparently misguided plan of care.^12^ Further details of the CDS design process are provided in eAppendix 4 in Supplement 2. The system did not operate during visits by patients in the control group.

**Table 1.** Prospectively Determined Outcomes Based on the Presenting Contextual Red Flag

Red flag (source)	Criteria	Outcome		
		Good (red flag documented to improve or resolve)	Bad (red flag documented to worsen or persist)	No change (red flag documented at same level or not documented as changed)
Uncontrolled chronic condition for which the patient is being treated (EHR, audio recording)	HbA_1c_ level has increased since prior measurement by >1 percentage point; SBP and/or DBP has increased since prior measurement by >10 mm Hg	Any decrease in HbA_1c_ level; any decrease in SBP or DBP	Any increase in HbA_1c_ level; any increase in SBP or DBP	No change in HbA_1c_ level; no change in SBP or DBP
Appointment nonadherence: clinics, laboratory tests, imaging, procedures (EHR, patient questionnaire, audio recording)	Missed ≥2 clinical encounters in past 4 mo; missed ≥1 laboratory tests and/or scheduled study in past 4 mo	Patient misses fewer appointments during the next 4 mo; patient completes scheduled laboratory tests and/or scheduled studies	Patient misses more appointments in the next 4 mo; patient completes fewer scheduled laboratory tests and/or scheduled studies	Patient misses same number of appointments in next 4 mo; patient misses same proportion of scheduled tests or studies
ED visits (EHR, patient questionnaire, audio recording)	≥2 ED visits in past 4 mo	Patient has fewer ED visits	Patient has more ED visits	Patient has same number of ED visits
Medication nonadherence (patient questionnaire, audio recording)	Answers “yes” to “Are you having any difficulty taking medications the way you have been told to take them?” and/or mentions it during visit	Patient takes medications as prescribed	Patient does not take medications as prescribed	Medical adherence not documented
Missed preventive care (patient questionnaire, audio recording)	Answers “yes” to “In the past 6 mo, have you declined any treatments, tests, or procedures that your provider recommended, like vaccines, blood tests, a colonoscopy?” and/or mentions it during visit	Patient receives recommended treatments, tests, or procedures	Patient does not receive recommended treatments, tests, or procedures	Patient receipt of treatments, tests, or procedures not documented
Weight gain or loss (audio recording)	≥4.5 kg (10 lb) gained or lost since last appointment	If overweight, weight is lower; if underweight, weight is higher	If overweight, weight is higher; if underweight, weight is lower	Patient weight stays the same
Unaware of diagnosis and/or results (audio recording)	Patient mentions that they are unaware of diagnosis or test results that should have been communicated to patient	Patient is aware of diagnosis or results	Patient is still unaware of diagnosis or results	Patient awareness or unawareness not documented
Difficulty with equipment (patient questionnaire, audio recording)	Unable to use or decline to use medical equipment, or using someone else’s equipment	Using own equipment	Not using (own) equipment	Patient use of equipment not documented
General statements by patients that are concerning such as “I’m not eating” (patient questionnaire, audio recording)	Individualized	Individualized and prospectively determined	Individualized and prospectively determined	Individualized, prospectively determined outcome not documented

Abbreviations: DBP, diastolic blood pressure; ED, emergency department; EHR, electronic health record; HbA_1c_, hemoglobin A_1c_; SBP, systolic blood pressure.

### End Points

The primary study end point constituted proportions of improved or resolved (vs worsened or no change) contextual red flags 6 months after the visit (Table 1). Secondary end points were the proportion of contextual red flags probed in the visit and the proportion of contextual factors identified during the visit that were addressed in the visit care plan (ie, the contextualization of care rate). The 9 categories of contextual red flags, along with subcategories, were developed in previous research.^13,14^ Exploratory outcomes were the effects of the EHR alerts on probing of contextual red flags that patients did not themselves bring up during the visit and the effect of the alerts on the red flags 6 months later.

Data were recorded using REDCap. After each encounter, research assistants (A.B.-C. and S.I.), who were blind to the assignment of each patient to the intervention or the control group, accessed the EHR and listened to the audio recording to identify contextual red flags (and determine whether each was probed by the clinician) and contextual factors (and determine whether each was incorporated into the care plan by the clinician), using the 4C methodology.^14,15^ Trained coders achieve approximately 85% interrater agreement about whether a care plan is contextualized or not.^15^ Consistent with the 4C methodology, red flags and contextual factors were noted by the coders from the patient questionnaire, EHR, or audio recording of the visit; factors noted on the audio recording emerged either as a result of clinician probing or spontaneous disclosure by the patient. For each red flag, coders prospectively defined the conditions under which the red flag would be considered improved or worsened in the future. Six months after each encounter, research assistants, again blind to the assignment of patients to the intervention or the control group, applied the predefined conditions to code each of the patients’ red flags from EHR review as improved, worsened, unchanged, or undocumented at follow-up.

### Sample Size

Based on prior studies,^6,16^ we assumed that contextual red flags with associated factors would be present in 50% of recorded visits, and that clinicians, unaided, would probe 50% of contextual red flags and contextualize care plans in 50% of visits with contextual factors. Based on the number of contextual red flags and factors we expected, we determined that to obtain 80% power to detect an absolute increase in contextualization rates when factors were present from 50% to 75%, we would need recordings from 192 intervention and 288 control patients (which also provides at least 80% power to detect the expected increase in probing).

### Randomization and Blinding

Patients were randomized 2:3 to either contextualized CDS (intervention) or usual care (control) using computer-generated randomization. We chose a ratio of 2:3 intervention to control patients because there would be fewer opportunities to contextualize care plans in the control group if fewer red flags were probed. Randomized assignments were available to research personnel responsible for activating the CDS tools immediately before the encounter, but those involved in coding visits and tracking outcomes were blinded. Although patients were blinded as to whether they were in the intervention or control group, it was not possible to blind clinicians because the intervention is a set of novel CDS tools that are not available in usual care.

### Statistical Analysis

We applied a logistic mixed-effects modeling approach to examine the impact of the study intervention. We fitted 2 models to the 6-month changes in the red flags. One model examined whether the red flag had improved (ie, resolved) (vs worsened or no change; primary end point); the other examined whether the red flag had worsened (vs improved or no change). Each included fixed effects of study group, study site, and whether the red flag’s associated factor had been incorporated into the care plan, and random effects of visit, patient, and clinician. As a sensitivity analysis, we also fitted a mixed ordered logit model to the outcomes categorized as worsened, no change or mixed, or improved.

For each red flag noted by coders, we fitted a model to whether the red flag was probed by the clinician (secondary end point). In addition to a fixed effect of study group (intervention vs control), we included fixed-effect covariates for study site, whether a relevant factor had been selected on the previsit questionnaire and whether the audio recorder was concealed or disclosed, and random effects of visit, patient, and clinician to adjust for clustering of red flags within visits and visits within clinicians and repeated visits by the same patient.

Similarly, for each contextual factor noted by 4C coders, we fitted a model to whether the factor was incorporated in the care plan by the clinician (secondary end point). In addition to a fixed effect of study group, we included fixed effects of study site; whether the factor was identified by probing of a red flag, selected in the patient questionnaire, or revealed spontaneously by the patient; whether the audio recorder was concealed or visible; and random effects of visit, patient, and clinician.

Our exploratory analyses of the impact of CDS on CCBs and alerts were limited to intervention visits with red flags that could be presented in the CCB (that is, red flags available before the visit itself), and examined whether the presence of a CCB or an alert for a red flag affected the likelihood of probing, incorporating a contextual factor in a plan if one was present, or having an improved or worsened red flag at 6 months, adjusting for site and random effects of visit, patient, and clinician. We also examined this question using all visits and including covariates for the intervention vs the control groups and the interaction of intervention vs control group and presence of CCB or alert (which could only happen in the intervention groups).

All analyses were performed on an intention-to-treat basis using R, version 3.6 (R Core Team). Two-sided *P* < .05 indicated statistical significance.

## Results

### Study Participants and Recruitment

From September 6, 2018, to March 4, 2021, 452 visits by adult patients (291 women [65.1%] and 156 men [34.9%]; mean [SD] age, 55.6 [15.1] years) with upcoming primary care appointments with 36 physicians and 3 advanced practice nurses (23 women [59.0%] and 16 men [41.0%]) completed the previsit questionnaire (431 [95.4%] with assistance) and carried a concealed audio recorder into their visit. Patient demographic characteristics were extracted from the clinical data warehouse at each site using patient identifiers. We compared race and ethnicity, along with other demographics, between the control and intervention groups to ensure that groups were balanced as to these characteristics, which have been previously associated with differences in health care. As shown in the Figure, 275 visits were randomized to the control group and 177 to the intervention group. Seven patients had 2 visits scheduled during the course of the study; each visit was individually randomized. Table 2 presents unique patient characteristics by study group at the first visit; patients did not differ between groups in demographic characteristics, numbers of red flags available before or during the visit, or whether they chose to reveal the audio recorder or keep it concealed. Table 3 provides physician demographic characteristics.

**Figure.**
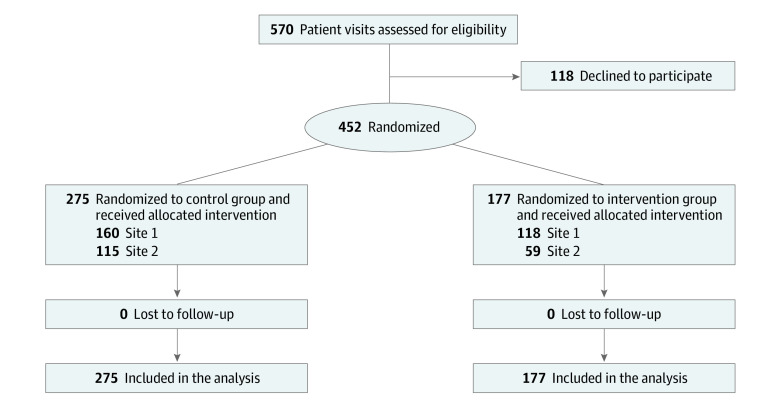
Study Flowchart Flow of patients through the clinical decision support intervention to prevent contextual errors trial.

**Table 2.** Participant Characteristics

Characteristic	Study group^a^		
	Control (n = 271)	Intervention (n = 176)	All (n = 447)
Site			
1	159 (58.7)	118 (67,0)	277 (62.0)
2	112 (41.3)	58 (33.0)	170 (38.0)
Sex			
Women	181 (66.8)	110 (62.5)	291 (65.1)
Men	90 (33.2)	66 (37.5)	156 (34.9)
Age, mean (SD), y^b^	55.6 (15.5)	55.6 (14.5)	55.6 (15.1)
Race and ethnicity^b^			
Asian	6 (2.2)	4 (2.3)	10 (2.3)
Black	132 (49.1)	79 (45.1)	211 (47.5)
Hispanic	41 (15.2)	30 (17.1)	71 (16.0)
White	72 (26.8)	52 (29.7)	124 (27.9)
Other^c^	18 (6.7)	10 (5.7)	28 (6.3)
No. of red flags per visit, mean (SD)	3.9 (2.4)	4.0 (2.3)	3.9 (2.4)
Only reported contextual red flag on patient questionnaire	78 (28.4)	57 (32.2)	135 (29.9)
Only had a contextual red flag that could be extracted from the EHR	33 (12.0)	18 (10.2)	51 (11.3)
Contextual red flag both reported on questionnaire and extracted from EHR	140 (50.9)	87 (49.1)	227 (50.2)
Revealed audio recorder			
Yes	49 (18.1)	28 (15.9)	77 (10.2)
No	222 (81.9)	148 (84.1)	370 (82.8)

Abbreviation: EHR, electronic health record.

^a^ Unless indicated otherwise, data are expressed as No. (%) of patients. Percentages have been rounded and may not total 100.

^b^ Data were not available for 2 control patients and 1 intervention patient.

^c^ Includes does not fit available categories, prefers not to answer, or unknown.

**Table 3.** Clinician Characteristics

Characteristic	Data (n = 39)^a^
Sex	
Women	23 (59.0)
Men	16 (41.0)
Race and ethnicity	
Asian	6 (15.4)
Black	5 (12.8)
Hispanic	4 (10.3)
White	23 (59.0)
Middle Eastern	1 (2.6)
Year of medical school graduation, median (IQR)	2003 (1994.5-2011.5)
Specialty clinic	
Family medicine	13 (33.3)
Internal medicine	26 (66.7)
Site	
1	26 (66.7)
2	13 (33.3)

^a^ Includes 36 physicians and 3 advanced practice nurses. Unless indicated otherwise, data are expressed as No. (%) of physicians.

### Outcomes

Table 4 summarizes the main outcomes of the study (in Supplement 2, eAppendix 5 presents raw study outcomes by group; Table 1 in eAppendix 6 presents regression models). For the primary end point, the study group did not have additional significant impact on outcomes beyond its impact on plans (adjusted odds ratio [aOR] for improvement, 0.96 [95% CI, 0.57-1.63; *P* = .88]; aOR for worsening, 1.31 [95% CI, 0.84-2.04; *P* = .86) (Table 4). However, across groups, contextualized plans were associated with a significantly higher likelihood of an improved red flag outcome (aOR, 2.13 [95% CI, 1,38-3.28]; *P* = .001) (Table 1 in eAppendix 6 in Supplement 2), and a significantly lower likelihood of a worsened red flag outcome (aOR, 0.67 [95% CI, 0.45-0.99]; *P* = .046). The ordered logit model produced the same conclusions (proportional odds aOR associated with contextualized plan, 1.76 [95% CI, 1.23-2.50]; *P* = .002).

**Table 4.** Patient Outcomes, Clinician Probing, and Clinician Contextual Care Planning by Study Group

	Unadjusted rate, No./No. (%)		Effect size, adjusted aOR (95% CI)^a^	*P* value
	Control group	Intervention group		
Outcome: improvement or resolution of red flags at 4-6 mo, adjusted for whether clinician incorporated contextual factor	247/509 (48.5)	163/383 (42.6)	0.96 (0.57-1.63)	.88
Probing: clinician probes contextual red flags	271/540 (50.2)	215/362 (59.4)	2.12 (1.14-3.93)	.02
Planning: clinician incorporates contextual factors into care plan	255/509 (50.1)	221/383 (57.7)	2.67 (1.32-5.41)	.006

Abbreviation: aOR, adjusted odds ratio.

^a^ All models were adjusted for random effects of visit, patient, and clinician and fixed effects of study site. The probing model also adjusted for whether the patient indicated a contextual factor on the previsit questionnaire and whether patient chose to make recorder visible to the clinician. The planning model also adjusted for whether the patient indicated a contextual factor on the previsit questionnaire, whether the patient chose to make recorder visible to the clinician, whether the clinician identified the contextual factor by probing a red flag, whether the patient spontaneously revealed the contextual factor, and the interaction between whether the clinician probed and whether the recorder was visible.

Physicians in the intervention group were more likely to probe red flags than physicians in the control group (aOR, 2.12 [95% CI, 1.14-3.93]; *P* = .02) (Table 4). In addition, red flags selected on the previsit questionnaire were less likely to be probed than those expressed orally during the visit (aOR, 0.15 [95% CI, 0.07-0.30]; *P* < .001) (Table 1 in eAppendix 6 in Supplement 2). There was no effect of whether the recorder was visible or concealed (aOR, 1.57 [95% CI, 0.63-3.96]; *P* = .34). eAppendix 7 in Supplement 2 shows the proportion of contextual red flags probed by a clinician, the proportion of contextual red flags for which a contextual factor was noted by the 4C coders, and the proportion of those contextual factors addressed in the care plan (ie, the contextualization of care rate by types of flags in the control and intervention groups). A list of 106 red flags in the other category is included in eAppendix 8 in Supplement 2. Overall, 215 of 362 contextual red flags (59.4%) were probed in the intervention group, compared with 271 of 540 (50.2%) in the control group. Contextual factors were identified in 196 of 362 (54.1%) in the intervention group compared with 258 of 540 (47.8%) of them in the control group, respectively. Overall, 156 of 196 contextual factors with presenting red flags (79.6%; 156 of all 383 contextual factors [40.7%]) were elicited in the intervention group compared with 180 of 258 (69.8%; 180 of all 509 contextual factors [35.4%]) in the control group.

Factors in the intervention group had a higher likelihood of incorporation into the care plan (aOR, 2.67 [95% CI, 1.32-5.41]; *P* = .006) (Table 4). As in past studies, factors identified through probing were additionally much more likely to be incorporated into care plans than those that were not (aOR, 61.65 [95% CI, 21.92-173.42]; *P* < .001) (Table 1 in eAppendix 6 in Supplement 2).^17^ Factors spontaneously revealed by patients were also more likely to be incorporated (aOR, 8.55 [95% CI, 3.98-18.36]; *P* < .001), but factors selected by patients on the previsit questionnaire were not (aOR, 1.42 [95% CI, 0.57-3.54]; *P* = .45). There was no effect of whether the recorder was visible or concealed (aOR, 0.86 [95% CI, 0.28-2.66]; *P* = .79). Figure 1 in eAppendix 9 in Supplement 2 summarizes the results of the models as a path analysis and illustrates the direct and indirect effects of the intervention on contextual probing, identification of contextual factors, contextualization of care plans, and patient outcomes.

### Impact of EHR Alerts

When the EHR populated the CCB or activated alerts for red flags before the start of the visit (ie, from the patient questionnaire or EHR triggers), the likelihood of the clinician probing the red flag increased significantly (aOR, 3.64 [95% CI, 1.18-11.20]; *P* = .02), and when a factor was present, the likelihood of incorporating it into the care plan also increased (aOR, 11.27 [95% CI, 2.27-55.85]; *P* = .003). In addition, when the red flag was the subject of an alert, it was less likely to have worsened 4 months later (aOR, 0.19 [95% CI, 0.05-0.73]; *P* = .02) but not more likely to have been improved (aOR, 0.75 [95% CI, 0.30-1.89]; *P* = .55), beyond the effect of whether the care plan had been contextualized. Tables 2 and 3 in eAppendix 6 in Supplement 2 present the regression coefficients for models exploring the effect of EMR CCB and alerts in intervention group visits and all visits, respectively.

## Discussion

In this randomized clinical trial, CDS tools did not improve patient outcomes; however, these tools increased the likelihood that a physician would address relevant patient life context in their care plan. As seen in previous studies, these contextualized care plans were more likely to result in improved prospectively defined patient health care outcomes.

Although it has been previously established that contextual errors are common, that they adversely affect patient outcomes, and that clinicians can learn to make fewer of them with feedback,^8^ this is the first study, to our knowledge, to demonstrate that CDS tools built into the EHR can decrease contextual errors. Just as CDS can guide biomedically focused decision-making by drawing on data from evidence-based guidelines and other forms of research evidence, it can also guide contextually informed decision making by drawing on data specific to the life circumstances and behaviors of individual patients.^18^

Contextualized CDS can serve as a corrective to biomedical bias in which clinicians prioritize biomedical information over contextual information, even when they have comparable implications for a patient’s clinical state.^7^ For instance, Weiner^19^ described a patient who had 3 unnecessary visits to the emergency department with acute illness because she missed her hemodialysis and no one asked her why. It turned out that she was trapped in a situation beyond her control that was easily resolved with the help of a social worker. Weiner and Schwartz^2^ have documented hundreds of similar examples.

The capacity of contextualized CDS to change outcomes is limited, to a varying degree, by the resources available to help patients and the contextual factor. In prior work, Binns-Calvey^20^ identified 12 broad contextual domains, with widely different resource needs. For instance, a patient struggling with a gap in knowledge or skills (eg, misunderstanding about how to use an inhaler) may need only a few minutes of the clinician’s time and guidance, whereas a patient with a transportation need may require assistance that only some clinics can provide.

The finding that physicians pay less attention to contextual red flags presented to them by CDS than those presented directly by the patient points to a limitation of this technology. The finding is consistent with prior work revealing that clinicians pay less attention to written compared with orally presented contextual information.^11^

Contextualized CDS has the advantage of being readily scalable. Future work should assess the effectiveness of the CDS when patients do not have the option to request assistance completing the questionnaire, because were they not to, the CDS would default to relying only on data culled from the medical record.

### Limitations

This study has some limitations. Although the disposition of some red flags, such as the numbers of missed appointments or glycated hemoglobin levels, could be coded objectively, other outcomes, such as a patient’s report of whether they are exercising more, are inherently subjective. Because coders are blinded as to study group, this limitation should not skew the results. It was not possible, however, to blind clinicians to study group because they can tell when they are receiving contextualized CDS.^21^ It is possible that clinicians try harder in the intervention, independent of the CDS. We attempted to mitigate any effect by explaining to clinicians that there would be no analysis of how they individually perform. Furthermore, the lack of an effect of the visible recorder on clinician probing suggests that they were not motivated to change their behavior just because they knew they were in a study.

Finally, the real effect of the intervention may be larger than what was found, because we had patients in both study groups complete the questionnaire, which may have primed those in the control group to raise contextual red flags and factors. We designed the study this way so we would not attribute impact to the CDS that was really from the questionnaire.

## Conclusions

Information about patients’ life circumstances and behaviors (ie, patient life context) is essential to identify and address in care planning to achieve desired outcomes; however, it is often overlooked. The findings of this randomized clinical trial support the use of CDS tools that draw on data elicited directly from the patient before the visit and from the EHR to facilitate decision-making that improves contextualization of care.

## Notes

Supplement 1.
Trial Protocol

Supplement 2. **eAppendix 1.** Portal Questionnaire **eAppendix 2.** Contextual Care Box **eAppendix 3.** Interruptive Alert **eAppendix 4.** CDS Design Process **eAppendix 5.** Raw Data **eAppendix 6.** Regression Tables **eAppendix 7.** Clinician Performance in Intervention vs Control **eAppendix 8.** Contextual Red Flags, “Other” Category **eAppendix 9.** Path Analytic Summary of Multivariate Models

Supplement 3.
Data Sharing Statement
