# Supplementary material for: Effect of Electronic Health Record Clinical Decision Support on Contextualization of Care: A Randomized Clinical Trial
Source: JAMA Netw Open. 2022 Oct 24;5(10):e2238231. doi: 10.1001/jamanetworkopen.2022.38231 (PMC9593230; doi:10.1001/jamanetworkopen.2022.38231)
Supplement: Supplement 1. — Trial Protocol [file jamanetwopen-e2238231-s001.pdf]

Integrating Contextual Factors into Clinical Decision Support to Reduce Contextual Error and Improve Outcomes in Ambulatory Care

Principal Investigator:

Saul Weiner, Professor, UIC Departments of Medicine and Pediatrics, sweiner@uic.edu

Co-Investigators:
Alan Schwartz, Professor, UIC Departments of Medical Education and Pediatrics,
alansz@uic.edu

William Galanter, Assistant Professor of Clinical Medicine, UIC Department of Medicine,
billg@uic.edu

Karl Kochendorfer, Chief Health Information Officer, UIC Department of Family Medicine,
kkoche1@uic.edu

Frances Weaver, Professor Professor, Public Health Sciences, Loyola University Chicago
fweaver@luc.edu

Aaron Michelfelder, Professor of Family Medicine, Bioethics & Health Policy, Loyola University Chicago
amichel@lumc.edu

Anita Varkey, Associate Professor of Medicine, Loyola University Chicago
anita.varkey@oakstreethealth.com

Study Location(s): UIC Primary Care Clinics, Loyola University Medical Center Primary Care Clinics

Sponsor: Agency for Health Care Quality and Research (R01HS25374)

Version: 2

Date: 6/8/2017

TABLE OF CONTENTS

Page

Table of Contents ... 2
List of Abbreviations ... 3
1.0 Project Summary/Abstract ... 4
2.0 Background/Scientific Rationale ... 6
3.0 Objectives/Aims ... 7
4.0 Eligibility ... 7
4.1 Inclusion Criteria ... 7
4.2 Exclusion Criteria ... 8
4.3 Excluded or Vulnerable Populations ... 8
5.0 Subject Enrollment ... 8
6.0 Study Design and Procedures ... 9
7.0 Expected Risks/Benefits ... 10
8.0 Data Collection and Management Procedures ... 11
9.0 Data Analysis ... 12
10.0 Data and Safety Monitoring ... 14
11.0 Regulatory Requirements ... 14
11.1 Informed Consent ... 15
11.2 Subject Confidentiality ... 15
11.3 Unanticipated Problems ... 16
12.0 References ... 17

LIST OF ABBREVIATIONS

4C	Content Coding for Contextualization of Care
CDS	Clinical Decision Support system
COI	Conflict of Interest
DHHS	Department of Health and Human Services
DMC	Data Monitoring Committee
DSMB	Data and Safety Monitoring Board
DSMP	Data and Safety Monitoring Plan
HIPAA	Health Insurance Portability and Accountability Act
ICD	Informed Consent Document
IRB	Institutional Review Board
LAR	Legally Authorized Representative
LUMC	Loyola University Medical Center
OHRP	Office of Human Research Protections
OPRS	Office for the Protection of Research Subjects
PHI	Protected Health Information
PI	Principal Investigator
RA	Research Assistant
SAE	Serious Adverse Event
USP	Unannounced Standardized Patient

1.0 Project Summary/Abstract

The term *patient context* refers to the myriad *contextual factors* in patients' lives that complicate the application of research evidence to patient care.[1] For instance, the inability of a patient to afford a medication for a particular condition is a contextual factor. Contextual factors can be addressed when correctly identified. Substituting a low cost generic for a high cost brand name medication may enable a patient to afford a medication. Addressing contextual factors in a care plan is termed *contextualizing care*.[2] Conversely, the failure to address a contextual factor when it is feasible to so is a *contextual error*, because it results in an inappropriate plan of care.[3] In sum, contextual errors are medical errors caused by inattention to patient context. They are common and linked to both diminished health care outcomes[4] and an increase in health care costs related to overuse and misuse of medical services.[5] These findings were determined using a validated method for coding audio recorded data called Content Coding for Contextualization of Care ("4C")[6] collected during the encounters by both real patients, and by unannounced standardized patients (USPs) employing checklists.[7]

Preventing contextual errors requires heightening clinician responsiveness to clues that there are contextual factors during the clinical encounter, in real time.[8, 9] These clues, termed *contextual red flags* are evident in two sources: the medical record and from patients directly.[10] An unexpected increase in glycosylated hemoglobin is an example of the former; a patient's comment that they've recently been having episodes of hypoglycemia reflects the latter. An effective intervention would prompt clinicians to determine whether there are underlying contextual factors that could be addressed in the care plan, averting contextual error. This desirable process is termed *contextual probing*.[6]

While clinical decision support (CDS) has been used to provide physicians with timely biomedical information at the point of care to prevent errors[11-13] and promote appropriate care,[14-16] this technology also affords an opportunity to draw physician attention to both contextual red flags and contextual factors in order to avert contextual errors. The proposed study is submitted in response to Special Emphasis Notice (SEN) NOT-HS-16-015, "Advancing the Collection and Use of Patient-Reported Outcomes and Patient Contextual Data to Improve Quality and Outcomes in Ambulatory Care through Health Information Technology." We will assess the potential of "contextualized CDS" to improve contextualization of care through a randomized controlled intervention trial, with assessment measures of both patient health care outcomes and averted costs associated with overuse and misuse of medical services. In addition to pursuing the aforementioned aim, the study design will adopt best practices for CDS design. We propose to implement highly personalized, concise, actionable contextual CDS strategies. The proposed study will pursue these aims by testing three hypotheses about contextualized CDS, and adhering to one design principle. The three hypotheses are that CDS:

1. *Reduces contextual error*: CDS tools that inform clinicians of contextual factors and prompt them to explore contextual red flags should result in a reduction in contextual error.
2. *Improve health care outcomes*: Contextualized CDS predicts improved health care outcomes defined as a partial or full resolution of the contextual red flag (e.g. elevated HgB A1c) after the index visit.
3. *Reduces avoidable health care costs*: Contextualized CDS is associated with a reduction in misuse and overuse of inappropriate or unnecessary medical services.

The design principle, referred to as *"Five Right"*[17] is to provide the right information to the right people through the right channels in the right format at the right point in care delivery.

To test the hypotheses, patients who consent to participate will be randomized to usual care or care enhanced with contextualized CDS. Participants will audio record their visits, and

the data will be coded using 4C. They will be followed for 4-6 months following the index visit for assessment of outcomes using an established tracking method.[6] In addition, USPs presenting with cases containing complicating contextual factors that if overlooked result in overuse and misuse of medical services, will be employed to assess the third hypothesis, and to supplement the data obtained by observing the effects of contextual alerts on the care of real patients for the first hypothesis.

2.0 Background/Scientific Rationale

A *contextual error* occurs when a care plan is inappropriate because of inattention to patient context.[1] Increasing the dosage of a patient's medication to manage deterioration of a chronic condition is a contextual error when the unaddressed underlying etiology is something in the patient's circumstances, such as a change in health insurance coverage, loss of social support or competing responsibilities. Contextual errors are a subtype of medical error as they reflect "….a wrong plan to achieve an aim."[3]

Our team has spent over a decade characterizing contextual errors (what they are and how to detect them), assessing their prevalence in various practice settings, measuring their impact on health care outcomes and costs, and trying to prevent them.[8,9] For the latter we have attempted medical education interventions,[4,18] and performance improvement strategies employing audit & feedback.[19] A common theme of all of this work has been that contextual errors occur when physicians overlook essential information about patients' circumstances and behaviors when planning their care, with measurably deleterious consequences for both health care outcomes and costs.[20] Reducing contextual error rates may require real time strategies, activated during the clinical encounter, that prompt physicians to explore and address patient context in care planning.

In our research employing real patient collected audio we learned that contextual errors are common. In a study in which 601 patients carried concealed audio recorders into their visits across multiple practice sites, we found that contextual red flags were present in 403 of visits (67%), and that contextual factors were revealed in 208, meaning that in 35% of encounters effective care required identifying and addressing a contextual factor.[4] Physicians were successful about 59% of the time, and responsible for a contextual error in the remaining 41%. In other words, about 14% (0.41 x 35%) of overall care was derailed by a contextual error. When we followed these patients for 9 months, the presenting problem at the time of the index visit was less likely to improve or resolve compared to visits without a contextual error (46% vs 71%; *P*= 0.002).

In our research employing unannounced standardized patients (USPs), actors presenting to clinicians as patients and collecting audio recordings, we documented similar performance problems, with high contextual error rates.[7] These errors are caused either by inattention to contextual red flags – i.e. not noticing or responding to clues of underlying contextual factors, or not addressing contextual factors in care planning. The cases we developed were designed such that physicians were also challenged to avoid making biomedical errors, e.g. overlooking evidence of gastroesophageal reflux in a patient with asthma presenting with increased symptoms after meals and when recumbent. Before deploying USPs, the cases were iteratively refined until board certified physicians reviewing paper based versions had a low probability of making either a biomedical or contextual error when explicitly informed of the contextual factor. [10] In situ, however, contextual error rates turned out to be both common and more frequent than biomedical errors. In a subsequent analysis we added up the direct service utilization costs of these errors using Medicare cost-based reimbursement data, by tabulating the expenses associated with misuse and overuse of medical services.[5] Over 400 encounters, biomedical errors contributed a mean cost of $30 per encounter, and contextual errors $231 per encounter (Figure 2).

Clinical Decision Support (CDS) provides a set of strategies for both individualizing and timing heightened awareness of patient specific information to inform decision making. CDS integrates patient specific data with a knowledge base and interprets the resulting data with clinical rules and guidelines to provide support to clinicians at various points in the care

process.[24] CDS can interact with clinicians in a variety of ways, from interactive alerts to passive visualization that guides decisions without interrupting clinicians. It can be real time, or a message that can come at a more convenient time for non-urgent information.[25]

To date the knowledge base in CDS systems has been primarily biomedical information, such as laboratory data, pharmaceuticals, diagnosis, patient allergies, age, sex, etc… We propose incorporating contextual information into the CDS knowledge base to allow CDS interventions that help clinicians pick up on contextual red flags and prevent contextual errors. The approach would embrace the “Five Rights” framework already widely adopted in CDS design.[17] CDS interventions must provide the *right information*, to the *right people*, through the *right channels,* in the right *intervention formats,* at the *right points* in workflow.

In the following section, we outline a plan for incorporating and rigorously assessing patient contextual information (contextual red flags and contextual factors) into CDS, and assessing its impact on contextual error rates, health care outcomes and the misuse and overuse of medical services, drawing on methods of measurement developed, validated and extensively employed in our prior research. In addition to measuring the benefits of contextualized CDS, this project will focus on best design practices, such that the contextual information is presented in a manner that is relevant to those who can act on the information and that results in the right action.

3.0 Objectives/Aims

We will assess the potential of “contextualized CDS” to improve contextualization of care through a 27 month randomized controlled intervention trial, with assessment measures of both patient health care outcomes and averted costs associated with overuse and misuse of medical services. In addition to pursuing the aforementioned aim, the study design will adopt best practices for CDS design. We propose to implement highly personalized, concise, actionable contextual CDS strategies. The proposed study will pursue these aims by testing three hypotheses about contextualized CDS, and adhering to one design principle. The three hypotheses are that CDS:

1. *Reduces contextual error*: CDS tools that inform clinicians of contextual factors and prompt them to explore contextual red flags should result in a reduction in contextual error.
2. *Improve health care outcomes*: Contextualized CDS predicts improved health care outcomes defined as a partial or full resolution of the contextual red flag (e.g. elevated HgB A1c) after the index visit.
3. *Reduces avoidable health care costs*: Contextualized CDS is associated with a reduction in misuse and overuse of inappropriate or unnecessary medical services.

4.0 Eligibility

- *Subjects include:*
  - 500 adult patients of primary clinics at UIC and Loyola Medical Center (LUMC) (we estimate approaching 1700 patients to recruit 500)
  - The clinicians (physicians or nurse practitioners) seeing the 500 patients (at least 20 clinicians and up to 200 clinicians)
  - Maximum subjects under this protocol: 2000 patients, 200 clinicians

4.1 Inclusion Criteria

- English-speaking adult patients presenting to outpatient primary care clinics for scheduled appointments who can be contacted in advance of their

appointment and the clinicians (physicians or nurse practitioners) seeing those patients at those visits.
- Eligible patients and their clinicians are identified from scheduled clinic appointments

4.2 Exclusion Criteria

- Patients with emergent or unscheduled visits or who do not speak English.

4.3 Excluded or Vulnerable Populations

- Patients who do not speak English are excluded because previously developed tools for assessing context and contextualization are only available in English and our 4C coding system has only been applied in English
- Clinician subjects include UIC and LUMC employees.

5.0 Subject Enrollment

- Clinicians: Clinicians will be informed of the study at their standing staff meetings. They'll be told that the purpose of the project is to assess whether enhanced clinical decision support, that provides both passive and actively delivered information provided by patients and extracted from their medication record about life challenges, or "contextual factors" that may be impacting their health care, can improve clinical decision making and health care outcomes and costs. They'll be informed that if they participate data collection will require listening in on the visit and that we will be inviting patients to audio record their visits. They'll also learn that this is a randomized study so that some of the time they'll see contextualized CDS information and other times they won't. They'll learn that they are not a unit of study, and we will be collecting no data about their individual performance. We'll also inform them that a decision not to participate will not impact their employment in any regard as we are a research team not connected to management. Those indicating they would like to participate will be contacted by an RA to complete the informed consent process

- Patients: The proposed protocol is that patients of participating physicians will be contacted about 2 weeks prior to a scheduled appointment to the adult primary care clinic at either of the two sites. Initial contact will be via mail with an opt out for a follow up phone call. If they don't opt out, the research assistant will call them. They will be informed that they are invited to participate in a study to determine whether providing their health care team with additional information in the electronic medical record about challenges or life circumstances they are facing that impact their care could improve the quality of their care, including their health outcomes. They will be informed that if they participate they will be asked and, assisted if needed, with completing a brief questionnaire for their medical record about challenges they are having that might impact their care. They'll also learn that when they arrive for their appointment they will receive a small digital audio record to carry into the visit. They'll be told that it is preferable to conceal the audio in their pocket or bag, but that they can take it out if they like. They'll be informed that their doctor supports the study. We also encourage all patients to turn off the audio recorder at any time if they change their mind about participation. Finally, they'll be informed that a member of the research team will access their record twice: first to note any information about their life situation that may be relevant to their health care now, and then several months later to see if key health care indicators noted at the visit have improved. Finally, they'll be told that their doctor may or may not receive the information they provided, based on random assignment. We have

allocated $20 to each patient participant and they'll be told that as well. Those who consent to participate will sign the consent document when they arrive for the appointment and are met by the RA. Only individuals who exhibit a full understanding of the protocol, and indicate they are comfortable recording their visit, are eligible to do so.

6.0 Study Design and Procedures

The protocol for the proposed trial is as follows: (a) Patients are contacted by phone approximately two weeks before a scheduled visit and invited to participate in a randomized controlled study of whether augmenting clinician attention to information about their life circumstances can result in higher quality care with better health care outcomes. (b) Among those who consent to participate, prior to randomization, subjects complete a brief questionnaire consisting of seven questions designed to elicit a broad range of contextual red flags, previously developed and validated with funding from another study (appendix). An affirmative response to any item prompts the respondent to then select one or more contextual factors if present. The instrument will be a commercially available portal tethered to the EHR for data transfer. Those who do not have web access will complete the instrument with the assistance of an RA over the phone before their visit or, if necessary, when they report for their appointment. These data upload (for both the intervention and control group) into the electronic medical record as discrete variables. (c) For those randomized to the intervention, these contextual factors along with contextual red flags already stored in the EHR will produce a variety of CDS, both passive and interruptive alerts. For visits by patients in the control group, the CDS system will not operate (d) Just prior to their appointment, in a private area near the waiting room, participants will receive a small encrypted digital audio recorder to conceal in a bag, or eye glass case or other common personal item. (e) As participants exit the visit, they return the audio recorder to an RA who uploads the audio to a secure server.

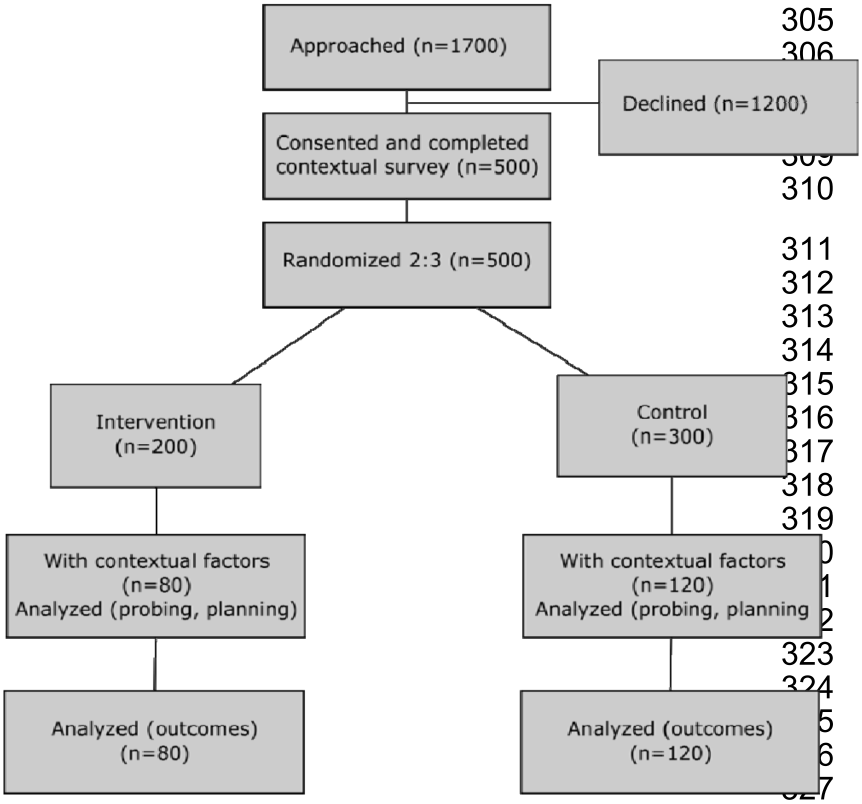

Figure 1: Participant flow diagram for randomized trial of contextualized clinical decision support in real patients.

Note that while patient encounters will be randomized, physicians will not. Participating physicians will provide care both with and without contextualized CDS. Hence there is no specific physician sample size required for this section of the study.

Randomization of unannounced standardized patients:

There are four reasons to employ USPs to assess the impact of an

intervention (contextualized CDS) on overuse and misuse of medical services. First, they are by definition standardized, meaning that physicians in both the control and intervention groups are seeing the "same" patient.[30] This experimental approach enables apples-to-apples comparisons (i.e. intrinsic risk adjustment) of clinical decision making, isolating the intervention as the sole changing variable. Second they assess actual performance in practice, rather than just skills.[22] The third reason is that USP cases can be designed around ambulatory presentations for which there is evidence based consensus about what constitutes appropriate care.[31] For instance, there is consensus that ordering radiographic studies on a patient presenting with uncomplicated lower back pain is an overuse of medical services. Similarly, ordering a malignancy work up on patient with weight loss in the setting of caloric deprivation is a misuse of medical services. The fourth reason is that USPs scripts can be customized around the particular CDS features we seek to assess. For instance, if we seek to assess whether alerts designed to inform clinicians when their patients are not adhering to medications in the setting of deteriorating chronic care management (e.g. a diabetic patients with elevated Accucheck readings in their log book) reduce unnecessary consultation of specialists, prescribing of additional medications etc…we will employ USP scripts that simulate such presentations.

For this project, 4 USP scripts with embedded contextual red flags and factors, drawn from our library of such cases will be selected. Their training and deployment will be managed by the UIC Graham Clinical Performance Center, which has extensive prior USP experience.[7,32] The scripts will be modified and customized to assess the efficacy of the selected CDS innovations such that failure of CDS to prevent inattention to contextual red flags or factors in USP cases would result in a contextual error. Following the development of the 4 scripts, each script will be portrayed at 10 control visits without CDS support and 10 intervention visits with CDS support, divided across the two sites, for a total of 80 USP visits.

	Year 1				Year 2				Year 3				Year 4			
**Recruitment and Randomization of Real Patients**																
Recruit 500 patients across two sites and assist with patient reported data entry (RA)																
Collect and 4C code audio recorded data (Project Manager and RA)																
Collect Outcomes Data based on tracking outcomes of contextual red flags on 120 patients for 6-9 months																
**Randomization and Deployment of USPs**																
Identify and train 13 USPs (CPC)																
Conduct 80 USP visits																
**Analysis**																
Data analyses of contextual error rates, outcomes, and costs of overuse and misuse of medical services																

Table 1: Project Timeline

7.0 Expected Risks/Benefits

7.1 Expected Risks

- Patients: The risks to patients are those that could be associated with any unintended dissemination of personal health information. A member of the research team, with patient consent, will access their medical record and will hear an audio recording of the patients encounter, collected by the patient. We have highly secure procedures and extensive experience avoiding any breach of PHI, using encryption for audio recorders, a secure server space approved for research data storage, and removal of patient identifiers when no longer needed for tracking outcomes (at about 4 months post index visit).
- Clinicians: The risks to clinicians are those associate with any harm to reputation if they perform poorly and the encounter, captured on audio, were disseminated. We use encrypted audio, with data transfer directly to a secure research server space, and removal of identifiers when no longer needed for tracking.

7.2 – Expected Benefits

- Patients: We are conducting this study because we have prior evidence that indicates that patient have better health care outcome when clinicians address patient context in care planning. We hypothesize that providing contextual information via CDS will increase contextualization of care. Those patients in the intervention group may therefore receive better care. Those in the control group may also benefit from the exercise of completing a brief questionnaire that primes them to consider how their life challenges are impacting their health care.
- Clinicians: Participating clinicians will benefit from clinical decision support that provides them with information about any life challenges patients in the intervention group are experiencing that may be relevant to care planning. In addition they'll receive CDS about how to use the information in care planning efficiently. For patients in the control group, clinicians will receive usual CDS.

8.0 Data Collection and Management Procedures

- Patients: Data for this study will come from 3 patients sources: (a) Their medical record. These are contextual red flags (e.g. missed appointments, loss of control of a chronic condition); (b) a patient completed inventory that is tethered to their electronic medical record, eliciting both contextual red flags and contextual factors that are not likely to be present in the EHR. See appendix for items; (c) Audio recordings they collect of their encounter, from which contextual red flags and contextual factors will be noted, and whether the care plan is contextualized or contextual errors are present. The extraction of all these data follow the Content Coding for Contextualization of Care ("4C") methodology as described in the proposal and previously published. These data will be accessible to the research assistant, project manager, and PI who are trained 4C coders, in a format that contains identifiers (MRNs). However, once they have extracted the data and paired data from the EHR with the audio coded data, identifiers are removed and replaced with arbitrary codes. They do retain, however, a crosswalk file between codes and MRNs separate from the research data so that they can follow up on patient chart based outcomes for the presenting red flag 4-6 months post index visit; once chart outcomes are extracted and tagged with the code, the crosswalk file will be destroyed. Beyond the coding team, data is only shared without identifiers. We will employ encryption on all audio recorders, and audio is immediately uploaded to a

secure research data approved server using a USB port following the visit. Access to the medical record is conducted by an RA trained in the "4C" method, as detailed in the proposal, which requires extracting specific information onto a spread – contextual red flags and factors as outlined in the research plan and detailed in our online and cited coding manual. In addition the patients note is linked to their data using a cross-walk file accessible only to the RA, project manager and PI, and then discarded after outcomes data is collected at 4-6 months, and identifiers are no longer needed.

- Clinicians: Encounters rather than clinicians are the unit of interest for this study. There will not be sufficient data collection from any individual clinician to draw inferences about his or her performance. In fact, clinicians are not randomized in this study. The same clinician will see patients in both the intervention and control groups, with and without contextualized CDS. He or she will also see USPs with and without clinical decision support. Hence, we plan only to collect aggregate data on the participating clinicians, including age range, years in practice, gender, and whether they are trained in internal medicine, family medicine or as advance practices nurses. As described directly above, the audio recordings by patients of their visit with their doctor or APN will be encrypted and stored on a secure server space. Their voices may be heard on audio, however, and recognizable. Encryption means that only the 4C coders and PI will have the capacity to hear the audio. As doctors are not a unit of study, we do not plan to keep the names of doctors associated with data from their visits. The audio files will be stored until the date of the completion of the study which will be four years from the start date. The digital files will then be irreversibly deleted.

9.0 Data Analysis

Hypothesis 1: CDS tools that inform clinicians of contextual factors and prompt them to ask questions when there are contextual red flags should result in a reduction in contextual error.

From real patient encounters (i.e. observational assessment)*:* Each visit is "Content Coded for Contextualization of Care" ("4C").[6] 4C coding consists of reviewing the medical record and listening to the audio to identify the presence or absence of each of the four steps to contextualize care: Are there contextual red flags? If so, did the clinician probe them? If so, did the patient reveal contextual factors? Note that patients sometimes reveal contextual factors without a probe.[33] Regardless, did the clinician incorporate the contextual factor(s) into the care plan? 4C enables care plans to be classified as either contextualized or inappropriate because of a contextual error. In the latter instance, 4C also pinpoints the cause of the error as either secondary to a failure to probe a contextual red flag or failure to incorporate a contextual factor into the care plan. Hence, 4C coding will ascertain whether contextualized CDS is associated with a reduction in contextual error. And, when contextual error rates are reduced it will ascertain whether the reduction is associated with a higher probing rate or a higher rate of addressing contextual factors revealed without a probe into the care plan.

From USP encounters (i.e. experimental assessment): Does not require 4C coding; instead we use checklists based on evidence based criteria for appropriate vs inappropriate care. We will recruit USPs to present with 4 different scripts, with counterbalancing of control vs. intervention EHR rules and specific USP scripts among physicians, so that each physician sees 2 intervention USPs with the contextualized CDS rules

active and 2 control USPs with no contextualized CDS. As in our past work, likelihood of probing contextual red flags and contextualizing care will be tested using mixed effects logistic regression models to control for case differences and clustering of cases within physicians.

Hypothesis 2: Contextualized CDS predicts improved health care outcomes defined as a resolution of the contextual error after the index visit.

This analysis is based exclusively on data generated from the real patient visits: 4C coding has been extensively utilized to track the resolution of contextual red flags. We've demonstrated in a research setting that contextualizing care does predict improved health care outcomes as defined by resolution or partial resolution of the presenting contextual red flag at 6-9 months following the index visit (with the range depending on timing of follow up visit or scheduled tests).[4] We propose to duplicate the methodology in this project, again tracking the status of the contextual red flags of patients seen at the index visit using a blind methodology, comparing those in the intervention group to the control. A detailed description of the process of scoring for outcomes based on contextualization of care is provided in the Content Coding for Contextualization of Care manual, publicly available.[27] As noted above, the outcome of interest is the disposition of the original contextual red flag when followed over time. The criteria for a good or poor outcome are prospectively determined, based on the original red flag, to avoid any bias resulting from knowledge of how the encounter gets coded. Determination of outcome is made blind to whether the index visit was coded as contextualized. A good outcome marks an improvement in the patient's condition as reflected in the contextual red flag. A poor outcome indicates no improvement in the contextual red flag.

Hypothesis 3: Contextualized CDS is associated with a reduction in misuse and overuse of medical services.

This analysis is based exclusively on data generated from the USP visits, in which physicians in the usual care and contextualized CDS groups see sets of "identical" patients for which misuse and overuse of medical services has been pre-defined. Utilizing our previously published methods,[5] we will adopt the economic perspective of the patient and their third party payer, if any, with a time horizon of the expected consequences of care during the 30 days following the consultation. We consider only the direct consequences of care associated with diagnosis or misdiagnosis. We will not consider downstream costs beyond the initial recommendations from the consultation, and we will not consider societal costs not incurred by the patient or payer, such as lost productivity. We will include only resources related to the immediate diagnostic and therapeutic management at the index visit. Resources are direct medical costs in the case of unnecessary treatment and foregone direct medical costs in the case of under treatment.

Sample size calculations

Real patients (see Figure 5): Assuming, based on our prior research[4] and data from the audit & feedback program,[19] that contextual red flags with associated factors will be present in 50% of recorded visits, that 30% of patients approached will consent to participate, that physicians unaided will probe 50% of contextual red flags and that physicians unaided will contextualize care in 50% of visits with contextual factors, we propose to power the study for 80% power to detect an absolute increase in probe rate from 50% to 75% with contextualized CDS, and a corresponding increase in contextualization rates from 50% to 75%. Testing hypothesis 1 requires 58 patients with identified contextual factors per group, and therefore at least 145 patients consented and recorded per group. Testing hypothesis 2 requires at least 60 contextualized care plans in the intervention group and 60 non-contextualized care plans in the control group, which we expect to achieve with 80 identified contextual factors in the

intervention group (requiring 192 recorded visits) and 120 identified contextual factors in the control group (requiring 288 recorded visits). Thus, to test all project hypotheses, we will approach and consent a sufficient number of patients (approximately 1600) to obtain recordings of 480 patients, randomize them to the intervention and control groups on a 2:3 basis (192 intervention, 288 control), and expect to identify contextual factors associated with red flags in 80 intervention and 120 control patients. As the primary care clinics at the participating sites see approximately 5,000 unique patients (UIC) and 25,000 (Loyola) annually, accrual is likely to require no more than 4-6 months.

Sample size (USPs): In our past work with USPs, physician made contextual errors approximately 80% of the time.[7] Assuming that the contextualized CDS enhances physician attention to red flags and leads them to probe substantially more often (e.g. increasing probe rate from 50% to 75%) and attend to identified information (e.g. increasing plan rate from 50% to 75%), we would expect overall contextual errors to occur no more than 45% of the time, and 28 control and 28 intervention USP visits would provide 80% power to detect such a difference and test hypothesis 1.

In our past work, we found an overall median cost of error of $194 when cases presented with contextual red flags, based on a median cost of $231 when contextual errors occurred and a median cost of $0 when contextual errors did not occur.[5] Based on bootstrapped simulation from our cost data in that study, 40 control and 40 intervention USP visits provide 83% power to detect the expected cost reduction (a median of $156) due to reduced contextual errors using a Wilcoxon rank-sum test with a significance level of p<.05. Accordingly, we will conduct 40 control and 40 intervention USP visits to provide sufficient power to test both study hypotheses. As the study comprises 4 USP visits (2 control, 2 intervention) per physician, we will recruit 20 physicians for this portion of the study.

10.0 Data and Safety Monitoring

We believe this study is minimal risk. However, to ensure the safety of research participants and to comply with NIH policies, a DSMB will be formed in early months of the project and given responsibility to review and approve study methods and analysis plan for the research. The DSMB will be organized by Dr. Weiner and will consist of senior, experienced clinicians and health services researchers. If deemed necessary by the IRB, the Principal Investigators will not be on the Data Safety and Monitoring Committee, thereby ensuring some level of independent review. When necessary, we will bring in experts from outside the project to serve on the committee. The DSMB will review interim data mid-way through the study using a predetermined stopping rule to determine whether the intervention group is being significantly benefitted (or harmed) over the control group and whether early stopping is necessary. In the rare event that an adverse event attributable to the CDS intervention is found, we will contact the patient’s provider and document in the chart the potential error that was found.

One mid-trial (half of patients enrolled) comparison of rate of 4-6 month post-visit resolution of visit contextual red flag for intervention vs. control visits using a mixed effects logistic regression model with random effect of clinic/site and fixed effect of trial arm. An effect of trial arm that is significant at the p<.01 level in either direction will trigger early stopping of additional recruiting (however, in patients already recruited who have completed the study visit, we will continue to obtain and analyze their 4-6 month post-visit medical records)

11.0 Regulatory Requirements

11.1 Informed Consent

- Patients: The proposed protocol is that patients will be contacted about 2 weeks prior to a scheduled appointment to the adult primary care clinic at either of the two sites. Initial contact will be via mail with an opt out for a follow up phone call. If they don't opt out, the research assistant will call them. They will be informed that they are invited to participate in a study to determine whether providing their health care team with additional information in the electronic medical record about challenges or life circumstances they are facing that impact their care could improve the quality of their care, including their health outcomes. They will be informed that if they participate they will be asked and, assisted if needed, with completing a brief questionnaire for their medical record about challenges they are having that might impact their care. They'll also learn that when they arrive for their appointment they will receive a small digital audio record to carry into the visit. They'll be told that it is preferable to conceal the audio in their pocket or bag, but that they can take it out if they like. They'll be informed that their doctor supports the study. We also encourage all patients to turn off the audio recorder at any time if they change their mind about participation. Finally, they'll be informed that a member of the research team will access their record twice: first to note any information about their life situation that may be relevant to their health care now, and then several months later to see if key health care indicators noted at the visit have improved. Finally, they'll be told that their doctor may or may not receive the information they provided, based on random assignment. We have allocated $20 to each patient participant and they'll be told that as well. Those who consent to participate will sign the consent document when they arrive for the appointment and are met by the RA. If a patient is unable to participate fully in the informed consent process, there will be delegation to a representative. Only individuals who exhibit a full understanding of the protocol, and indicate they are comfortable recording their visit, are eligible to do so.

- Clinicians: Clinicians will be informed of the study at their standing staff meetings. They'll be told that the purpose of the project is to assess whether enhanced clinical decision support, that provides both passive and actively delivered information provided by patients and extracted from their medication record about life challenges, or "contextual factors" that may be impacting their health care, can improve clinical decision making and health care outcomes and costs. They'll be informed that if they participate data collection will require listening in on the visit and that we will be inviting patients to audio record their visits. They'll also learn that this is a randomized study so that some of the time they'll see contextualized CDS information and other times they won't. They'll learn that they are not a unit of study, and we will be collecting no data about their individual performance. We'll also inform them that a decision not to participate will not impact their employment in any regard as we are a research team not connected to management. Those indicating they would like to participate will be contacted by an RA to complete the informed consent process.

11.2 Subject Confidentiality

- Patients: Data for this study will come from 3 patient sources: (a) Their medical record. These are contextual red flags (e.g. missed appointments, loss of control of a chronic condition); (b) a patient completed inventory that is tethered to their electronic medical record, eliciting both contextual red flags and contextual factors

that are not likely to be present in the EHR. See appendix for items; (c) Audio recordings they collect of their encounter, from which contextual red flags and contextual factors will be noted, and whether the care plan is contextualized or contextual errors are present. The extraction of all these data follow the Content Coding for Contextualization of Care ("4C") methodology as described in the proposal and previously published. These data will be accessible to the research assistant, project manager, and PI who are trained 4C coders, in a format that contains identifiers (MRNs). However, once they have extracted the data and paired data from the EHR with the audio coded data, identifiers are removed and replaced with arbitrary codes. They do retain, however, a crosswalk file between codes and MRNs separate from the research data so that they can follow up on patient chart based outcomes for the presenting red flag 4-6 months post index visit; once chart outcomes are extracted and tagged with the code, the crosswalk file will be destroyed. Beyond the coding team, data is only shared without identifiers. We will employ encryption on all audio recorders, and audio is immediately uploaded to a secure research data approved server using a USB port following the visit. Access to the medical record is conducted by an RA trained in the "4C" method, as detailed in the proposal, which requires extracting specific information onto a spread – contextual red flags and factors as outlined in the research plan and detailed in our online and cited coding manual. In addition, the patients note is linked to their data using a cross-walk file accessible only to the RA, project manager and PI, and then discarded after outcomes data is collected at 4-6 months, and identifiers are no longer needed.

- Clinicians: Encounters rather than clinicians are the unit of interest for this study. There will not be sufficient data collection from any individual clinician to draw inferences about his or her performance. In fact, clinicians are not randomized in this study. The same clinician will see patients in both the intervention and control groups, with and without contextualized CDS. He or she will also see USPs with and without clinical decision support. Hence, we plan only to collect aggregate data on the participating clinicians, including age range, years in practice, gender, and whether they are trained in internal medicine, family medicine or as advance practices nurses. As described directly above, the audio recordings by patients of their visit with their doctor or APN will be encrypted and stored on a secure server space. Their voices may be heard on audio, however, and recognizable. Encryption means that only the 4C coders and PI will have the capacity to hear the audio. As doctors are not a unit of study, we do not plan to keep the names of doctors associated with data from their visits.

11.3 Unanticipated Problems

- Unanticipated problems will be reported to the UIC IRB and the DSMB, as well as to the sponsor if required by conditions of the grant.

12.0 References

1. Weiner SJ. Contextualizing medical decisions to individualize care: lessons from the qualitative sciences. *J Gen Intern Med.* 2004;19(3):281-285.
2. Weiner SJ. From research evidence to context: the challenge of individualizing care. *ACP J Club.* 2004;141(3):A11-12.
3. Weiner SJ. Contextual Error. In: Kattan M, ed. *Encyclopedia of Medical Decision Making*: SAGE; 2009:198-202.
4. Weiner SJ, Schwartz A, Sharma G, et al. Patient-centered decision making and health care outcomes: an observational study. *Ann Intern Med.* 2013;158(8):573-579.
5. Schwartz A, Weiner SJ, Weaver F, et al. Uncharted territory: measuring costs of diagnostic errors outside the medical record. *BMJ Qual Saf.* 2012.
6. Weiner SJ, Kelly B, Ashley N, et al. Content Coding for Contextualization of Care: Evaluating Physician Performance at Patient-Centered Decision Making. *Med Decis Making.* 2013.
7. Weiner SJ, Schwartz A, Weaver F, et al. Contextual errors and failures in individualizing patient care: a multicenter study. *Ann Intern Med.* 2010;153(2):69-75.
8. Weiner SJ SA. Contextual Errors in Medical Decision Making: Overlooked and Understudied. *Acad Med.* 2016;91(5):657-662.
9. Weiner SJ, Schwartz A. *Listening for what matters : avoiding contextual errors in health care.* Oxford ; New York: Oxford University Press; 2016.
10. Weiner SJ, Schwartz A, Yudkowsky R, et al. Evaluating physician performance at individualizing care: a pilot study tracking contextual errors in medical decision making. *Med Decis Making.* 2007;27(6):726-734.
11. Galanter WL, Bryson ML, Falck S, et al. Indication alerts intercept drug name confusion errors during computerized entry of medication orders. *PLoS One.* 2014;9(7):e101977.
12. Galanter W, Falck S, Burns M, Laragh M, Lambert BL. Indication-based prescribing prevents wrong-patient medication errors in computerized provider order entry (CPOE). *Journal of the American Medical Informatics Association : JAMIA.* 2013;20(3):477-481.
13. Galanter WL, Didomenico RJ, Polikaitis A. A trial of automated decision support alerts for contraindicated medications using computerized physician order entry. *Journal of the American Medical Informatics Association : JAMIA.* 2005;12(3):269-274.
14. Galanter WL, Thambi M, Rosencranz H, et al. Effects of clinical decision support on venous thromboembolism risk assessment, prophylaxis, and prevention at a university teaching hospital. *American journal of health-system pharmacy : AJHP : official journal of the American Society of Health-System Pharmacists.* 2010;67(15):1265-1273.
15. Galanter WL, Polikaitis A, DiDomenico RJ. A trial of automated safety alerts for inpatient digoxin use with computerized physician order entry. *Journal of the American Medical Informatics Association : JAMIA.* 2004;11(4):270-277.
16. Kucher N, Koo S, Quiroz R, et al. Electronic alerts to prevent venous thromboembolism among hospitalized patients. *N Engl J Med.* 2005;352(10):969-977.
17. Sirajuddin AM, Osheroff JA, Sittig DF, Chuo J, Velasco F, Collins DA. Implementation pearls from a new guidebook on improving medication use and outcomes with clinical decision support. Effective CDS is essential for addressing healthcare performance improvement imperatives. *J Healthc Inf Manag.* 2009;23(4):38-45.
18. Schwartz A, Weiner SJ, Harris IB, Binns-Calvey A. An educational intervention for contextualizing patient care and medical students' abilities to probe for contextual issues in simulated patients. *JAMA.* 2010;304(11):1191-1197.

19. Weiner SJ SA, Sharma G, Binns-Calvey A, Ashley N, Kelly B, Weaver FM. Patient collected audio for performance assessment of the clinical encounter. *Jt Comm J Qual Patient Saf.* 2015;42(6):273-278.
20. Weiner SJ, Schwartz A, Weaver F, et al. Overlooking Contextual Information When Individualizing Care: A Source of Medical Error and Avoidable Cost. *Journal of General Internal Medicine.* 2009;24:130-130.
21. Weiner SJ, Schwartz A. Directly Observed Care: Can Unannounced Standardized Patients Address a Gap in Performance Measurement? *J Gen Intern Med.* 2014.
22. Schwartz A, Weiner SJ, Binns-Calvey A. Comparing announced with unannounced standardized patients in performance assessment. *Jt Comm J Qual Patient Saf.* 2013;39(2):83-88.
23. Ivers N, Jamtvedt G, Flottorp S, et al. Audit and feedback: effects on professional practice and healthcare outcomes. *Cochrane Database Syst Rev.* 2012(6):CD000259.
24. Beeler PE, Bates DW, Hug BL. Clinical decision support systems. *Swiss Med Wkly.* 2014;144:w14073.
25. Osheroff JA, Healthcare Information and Management Systems Society. *Improving outcomes with clinical decision support : an implementer's guide.* 2nd ed. Chicago, IL: HIMSS; 2012.
26. Dawson J, Doll H, Fitzpatrick R, Jenkinson C, Carr AJ. The routine use of patient reported outcome measures in healthcare settings. *BMJ.* 2010;340:c186.
27. Weiner S, Ashley N, Binns-Calvey A, Kelly B, Sharma G, Schwartz A. . Content Coding for Contextualization of Care. Version 6.0. Harvard Dataverse Network Project [on line]. December 2014.
28. Phansalkar S, Desai A, Choksi A, et al. Criteria for assessing high-priority drug-drug interactions for clinical decision support in electronic health records. *BMC Med Inform Decis Mak.* 2013;13(1):65.
29. Tilson H, Hines LE, McEvoy G, et al. Recommendations for selecting drug-drug interactions for clinical decision support. *American journal of health-system pharmacy : AJHP : official journal of the American Society of Health-System Pharmacists.* 2016;73(8):576-585.
30. Srinivasan M, Franks P, Meredith LS, Fiscella K, Epstein RM, Kravitz RL. Connoisseurs of care? Unannounced standardized patients' ratings of physicians. *Med Care.* 2006;44(12):1092-1098.
31. Peabody JW, Luck J, Glassman P, Dresselhaus TR, Lee M. Comparison of vignettes, standardized patients, and chart abstraction: a prospective validation study of 3 methods for measuring quality. *JAMA.* 2000;283(13):1715-1722.
32. Dr. Allan L. and Mary L. Graham Clinical Performance Center. http://chicago.medicine.uic.edu/grahamcpc/.
33. Schwartz A, Weiner SJ, Binns-Calvey A, Frances M. Providers contextualise care more often when they discover patient context by asking: meta-analysis of three primary data sets. *Bmj Quality & Safety.* 2016;25(3):159-163.
