# Supplementary material for: Effect of Electronic Health Record Clinical Decision Support on Contextualization of Care: A Randomized Clinical Trial
Source: JAMA Netw Open. 2022 Oct 24;5(10):e2238231. doi: 10.1001/jamanetworkopen.2022.38231 (PMC9593230; doi:10.1001/jamanetworkopen.2022.38231)
Supplement: Supplement 2. — eAppendix 1. Portal Questionnaire eAppendix 2. Contextual Care Box eAppendix 3. Interruptive Alert eAppendix 4. CDS Design Process eAppendix 5. Raw Data eAppendix 6. Regression Tables eAppendix 7. Clinician Performance in Intervention vs Control eAppendix 8. Contextual Red Flags, “Other” Category eAppendix 9. Path Analytic Summary of Multivariate Models [file jamanetwopen-e2238231-s002.pdf]

Supplementary Online Content

Weiner SJ, Schwartz A, Weaver F, et al. Effect of electronic health record clinical decision support on contextualization of care: a randomized clinical trial. *JAMA Netw Open*. 2022;5(10):e2238231. doi:10.1001/jamanetworkopen.2022.38231

eAppendix 1. Portal Questionnaire
eAppendix 2. Contextual Care Box
eAppendix 3. Interruptive Alert
eAppendix 4. CDS Design Process
eAppendix 5. Raw Data
eAppendix 6. Regression Tables
eAppendix 7. Clinician Performance in Intervention vs Control
eAppendix 8. Contextual Red Flags, “Other” Category
eAppendix 9. Path Analytic Summary of Multivariate Models

This supplementary material has been provided by the authors to give readers additional information about their work.

© 2022 Weiner SJ et al. *JAMA Network Open*.

eAppendix 1. Portal Questionnaire

Patient Portal Questionnaire on Contextual Red Flags and Factors

Please take a few minutes to complete this questionnaire. The information you provide may be directly available to your doctor at the time of your upcoming visit

1. **Patients often have many medications to take at different times during the day. Are you having any difficulty taking medications the way you have been told to take them?** *(Please select one.)*

☐**YES** ☐**NO**

IF YOU ANSWERED "YES" – PLEASE CHECK ALL THE BOXES THAT APPLY:

☐ **I have other things I need to do that interfere with taking my medication.**
(For example: I can't take breaks during my job to take medication; I am taking care of a sick familymember.)

☐ **I don't have anyone to help me take my medication correctly.**
(For example: I need someone to help measure insulin dosage; I need someone to put mymedication in a daily pill box.)

☐ **I didn't have or ran out of my medication.**
(For example: My medication didn't arrive from the pharmacy; I didn't have medication with me at the time to take.)

☐ **Another doctor/nurse gave me different instructions for taking my medication.My medication is too expensive**.
(For example: I can't pay for the medication.)

☐ **I sometimes forget to take or am unsure how to take my medication.**
(for example: I am confused about when to take my medication; OR I didn't know if I should take my medication on anempty stomach.)

☐ **I'm not sure how to order my medication refills.**

☐ **I've been so overwhelmed or sad at times that I haven't been able to take my medication as prescribed.**

☐ **It goes against my beliefs or culture to take medicine.**
(For example: In my religion, we do not take medications when we are sick.)

☐ **My living situation interferes with my ability to take my medication correctly.**
(For example: I don't have a safe place to keep my medications.)

☐ **I don't think I need to take medicine.**

☐ **Other (Limited to 100 letters and spaces): ________________________________**

© 2022 Weiner SJ et al. *JAMA Network Open*.

2. **Patients often have many appointments, labs and other tests, as well as scheduled phonecalls. Are you having difficulty keeping any of these appointments?** *(Please select one.)*

☐**YES** ☐**NO**

[If YES select]:

Which of these are you having difficulty keeping. Check all that apply:

☐ **Appointments**

☐ **Labs and other tests**

☐ **Schedule phone calls**

IF YOU ANSWERED "YES" – PLEASE CHECK ALL THE BOXES THAT APPLY:

☐ **I have other things I need to do that interfere with keeping my appointments.**
(For example: I can't take time off from my job to come in for an appointment; I am taking care ofa sick family member.)

☐ **I had trouble getting to my appointments.**
(For example: There's no public transportation where I live that I can afford; I need someone tobring me to my appointment.)

☐ **I wasn't sure or forgot when my appointments were.I saw another doctor/nurse instead.**

☐ **Nobody scheduled the appointment or test for me and/or I didn't know how to schedule theappointment.**

☐ **I'm not sure why I was supposed to have these appointments.I didn't think I needed to keep these appointments.**

☐ **I've been so overwhelmed or sad at times that I hadn't been able to keep my appointments.It goes against my beliefs or culture to see a doctor/nurse.**

☐ **My living situation interferes with my ability to keep appointments.**

☐ **I don't think I need these tests or appointments.**

☐ **Other (Limited to 100 letters and spaces): ___________________________________**

3. **In the past six months, have you declined any treatments, tests, or procedures that yourprovider recommended; like vaccines, blood tests, a colonoscopy?** *(Please circle one.)*

☐**YES** ☐**NO**

[If YES select]:

Which of these are you having difficulty keeping. Check all that apply:

☐ **Vaccines**

☐ **Blood tests**

☐ **A colonoscopy or other procedure**

© 2022 Weiner SJ et al. *JAMA Network Open*.

IF YOU ANSWERED "YES" – PLEASE CHECK ALL THE BOXES THAT APPLY:

☐ **I have other things I need to do that interfere with getting the recommended treatment, test,and/or procedure.**
(For example: I can't take off from my job to come in for a test; I am taking care of a sick family member.)

☐ **I don't have anyone to help me with the recommended treatment, test, and/or procedure.**
(For example: I need someone to pick me up after a colonoscopy.)

☐ **I had trouble getting to where I needed to be.**
(For example: I did not have transportation.)

☐ **I received the treatment, test, or procedure somewhere else.**
☐ **The treatments, tests or procedures are too expensive.**
☐ **I'm not sure what the treatment, test, or procedure is for.**
☐ **I've been so overwhelmed or sad at times that I haven't been able to get the treatment, test, or procedure.**
☐ **It goes against my beliefs or culture to get the treatment, test, or procedure.**
☐ **My living situation interferes with my ability to get the treatment, test, or procedure.**
☐ **I don't want to know the results of these tests.**

☐ **I think you can get sick from some of these treatments, tests, or procedures or they can be painful or uncomfortable.**
(For example: The flu shot can make you sick.)

☐ **I don't believe I need these treatments, tests, or procedures.**
☐ **Other (Limited to 100 letters and spaces): ______________________________**

4. Have you visited an Emergency Room or Urgent Care clinic more than once in the past six months? *(Please select one.)*

☐ **YES** ☐ **NO**

IF YOU ANSWERED "YES" – PLEASE CHECK ALL THE BOXES THAT APPLY:

☐ **I have other things I need to do that interfere with my receiving routine care.**
(For example: I can't take off from work to come in for an appointment; I am taking care of a sickfamily member.)

☐ **I don't have anyone to take me to the clinic during regular hours.**
(For example: I need someone to drive me to the clinic.)

☐ **I couldn't get in to see my doctor/nurse quickly enough.**
☐ **I didn't have transportation to get to the clinic for care during regular hours.**
☐ **Regular clinic visits are too expensive.**
☐ **I'm not sure how to schedule an appointment during regular clinic hours.**

© 2022 Weiner SJ et al. *JAMA Network Open*.

☐ **I've been so overwhelmed or sad at times that I couldn't make it to the clinic during regularhours.**

☐ **My living situation interferes with my ability to make it to the clinic during regular hours.**

☐ **I didn't want to admit something was wrong and waited too long.**

☐ **I didn't want to bother my regular doctor/nurse.**

☐ **The choices I make in my life have caused me to use the ER.** (for example: risky or illegal behaviors).

☐ **Other (Limited to 100 letters and spaces): ________________________________**

5. Are you having any difficulty with your medical equipment? For example: a glucometer to test your blood sugar, a CPAP machine for sleep apnea, a wheelchair. *(Please select one.)*

☐**YES** ☐**NO**

IF YOU ANSWERED "YES" – PLEASE CHECK ALL THE BOXES THAT APPLY:

☐ **I have other things I need to do that interfere with using my equipment.** (For example: I can't use my equipment at work. I don't have time to use the equipment becauseI am talking care of family members).

☐ **I don't have anyone to help me with my equipment.** (For example: I need someone to help load and unload my wheelchair from a car.)

☐ **I never received the equipment.**

☐ **My equipment is broken.**

☐ **The equipment is too expensive.**

☐ **I'm not sure I know how to use the equipment.**

☐ **It is complicated to work the equipment.**

☐ **I am embarrassed to use my equipment in front of others.**

☐ **I've been so overwhelmed or sad at times that I haven't been able to use my equipment.**

☐ **It goes against my beliefs or culture to use medical equipment.**

☐ **My living situation interferes with my ability to use my equipment.** (For example: My home is too small to use a wheelchair.)

☐ **I don't want to rely on equipment.**

☐ **I don't believe I need to use the equipment.**

☐ **Other (Limited to 100 letters and spaces): ________________________________**

6. Are you having difficulties carrying out the activities important to stay healthy, such as exercising, eating healthy food, or checking your blood pressure or blood sugar levels,

© 2022 Weiner SJ et al. *JAMA Network Open*.

dependingon your medical conditions?

☐ **YES** ☐ **NO**

IF YOU ANSWERED "YES" – PLEASE CHECK ALL THE BOXES THAT APPLY:

☐ **I have other things I need to do that interfere with managing my health.**
(For example: I can't take breaks during my job to check my blood sugar; I am taking care of a sickfamily member.)

☐ **I don't have anyone to help me.**
(For example: I need someone to help me use my glucometer to check my blood sugar level.)

☐ I **can't get what I need to manage my health.**
(For example: I can't get healthy food because there are no grocery stores in my neighborhood; Ican't check my blood pressure because I don't have a home blood pressure monitor.)

☐ **Another doctor/nurse told me to do something different.**
☐ **Some of the things I need to manage my health are too expensive.**
(For example: Healthy foods are expensive.)

☐ **I sometimes forget or am unsure what I'm supposed to do to manage my health.**
☐ **I'm not sure what I'm supposed to do to manage my health.**
☐ **I'm not sure why I'm supposed to manage my health.**
☐ **I felt better and didn't think I needed to follow the plan.**
☐ **I've been so overwhelmed or sad at times that I haven't been able to manage my health.**
☐ **It goes against my beliefs or culture to follow the plan.**
☐ **My living situation interferes with my ability to manage my health.**
(For example: It's hard to eat healthy foods when others in my house do not.)

☐ **My doctor and I don't always agree on what I should do for my health.**
☐ **Other (Limited to 100 letters and spaces): _________________________**

7. Is there anything going on in your life that is affecting the way you manage your health that you'd like your doctor to know about?

(Limited to 100 letters and spaces): _______________________________________

© 2022 Weiner SJ et al. *JAMA Network Open*.

eAppendix 2. Contextual Care Box

Contextual Care Box Example

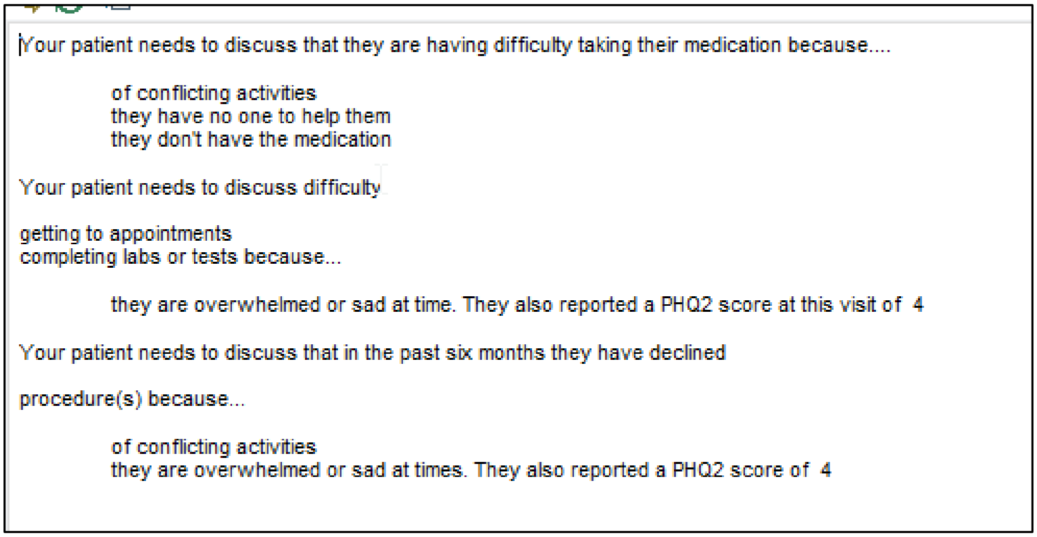
Your patient needs to discuss that they are having difficulty taking their medication because....

of conflicting activities
they have no one to help them
they don't have the medication

Your patient needs to discuss difficulty

getting to appointments
completing labs or tests because...

they are overwhelmed or sad at time. They also reported a PHQ2 score at this visit of 4

Your patient needs to discuss that in the past six months they have declined

procedure(s) because...

of conflicting activities
they are overwhelmed or sad at times. They also reported a PHQ2 score of 4

eAppendix 3. Interruptive Alert

Interruptive Alert Example:

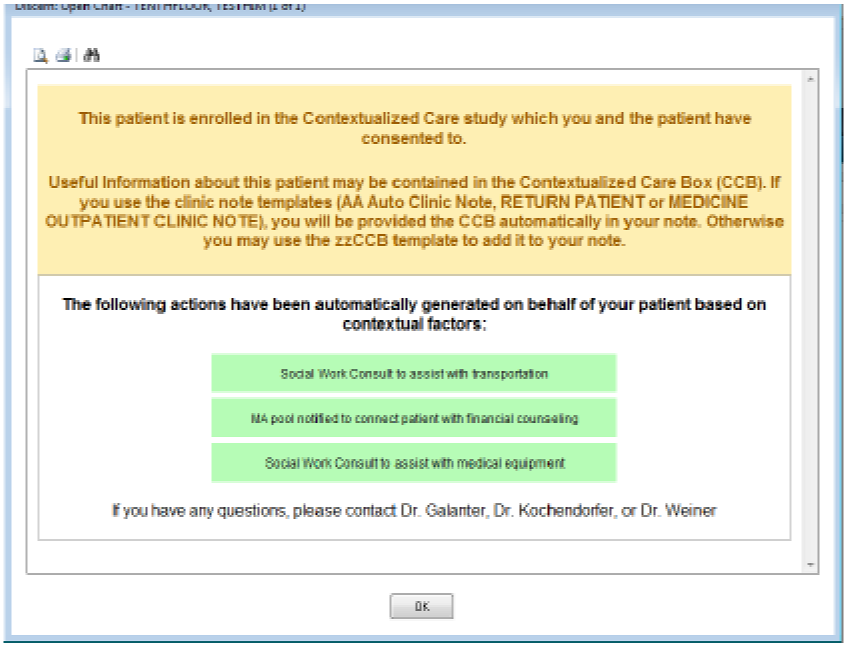

eAppendix 4. CDS Design Process

The clinical decision support (CDS) design was supervised by WG who has substantial experience designing CDS, specifically in Cerner. He was assisted by clinical informatics fellows, the rest of the research team, and IS staff at UIC. For the Epic site, the design in Cerner was adapted as needed by the research team at Loyola with assistance from IS staff.

The CDS design process began by building a table to describe, for each item elicited from a patient (i.e., a contextual red flag, including the contextual factor they selected for the red flag in the drop-down) and for selected data in the EHR, what the CDS intervention would be and the logic used to trigger the CDS. There were four types of CDS used: 1) production of the "Contextual Care Box" (CCB) which listed out all passive suggestions to clinicians in text when starting a note (supplement B); 2) An alert which displayed at the beginning of all study sessions which, among other things, listed out the information in the CCB (supplement C); 3) interruptive alerts that were supplied sparingly to clinicians, e.g., to to re-direct an apparently misguided plan of care (such as prescribing an unnecessarily expensive medication to a patient who has indicated they cannot afford their medication); and 4) orders or consults placed automatically without interrupting clinicians, such as referral to social work for a patient needing assistance with transportation.

Each intervention in the table was described in the CCB column or CDS (non CCB) column. Each non-CCB had a separate flowsheet of a rule associated with the CDS, which was standard at Site 1 for all CDS. Table 1 provides an example of a CCB message, an interruptive alert, and a non-interruptive placement of a consult (one of each highlighted).

Table 1: Example of the design process for CDS responses to patient questionnaire.

	Contextual Red Flag	Contextual Factor	Contextual Care Box (CCB)	CDS (non CCB)	Desired Intervention
1b	**Patient having difficulty taking medications the way they have been told to take them.**	*I don't have anyone to help me take my medication correctly.*	*…they have no one to help them*.	Interruptive alert with options for: order social work consult, Pharmacy Consult and/or arrange for a visiting nurse.	Provider and patient discuss and arrive at customized plan.
3e	**Patient reports that in the past six months they have declined a treatments, tests, or procedure that their provider recommended,**	*The treatments, tests or procedures are too expensive.*	…they are too expensive	Social work consult to assist insurance coverage.	Arrive at customized plan.

© 2022 Weiner SJ et al. *JAMA Network Open*.

	Contextual Red Flag	Contextual Factor	Contextual Care Box (CCB)	CDS (non CCB)	Desired Intervention
	such as a vaccine, blood tests, or colonoscopy.				

The rule for the interruptive alert in 1b of the table is shown below:

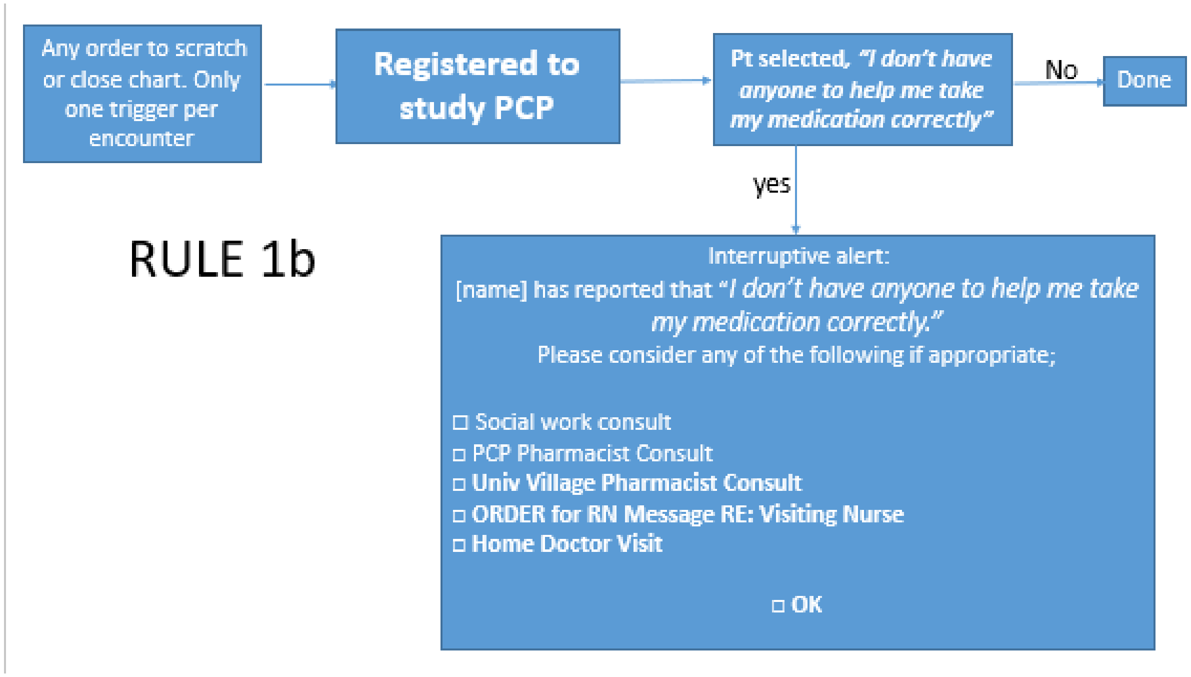

A similar process was employed for the design of the CDS based on data in the EHR which was not derived from the patient questionnaire. CDS, starting with the construction of a table showing, for each red flag, what CDS action should occur, what clinician intervention should follow (if the CDS is successful), and a description of the rule. Again, we've included (Table 2) one rule which adds a comment to the CCB, one rule which produces an automatic action, and one rule with produces an interruptive alert (one of each highlighted).

© 2022 Weiner SJ et al. *JAMA Network Open*.

Table 2: Example of the design process for CDS responses to EHR data.

	Contextual Red Flag	CCB	CDS (non-CCB)	Desired Intervention	Rules Description
E4	**Loss of Control of Diabetes: HgB A1c has increased since prior measurement by > 1% point**	*Your patient has a glycosylated hemoglobin that has risen by > 1% point*		Identify contextual factor(s) and arrive at customized plan.	If HbA1C (lab or POCT) in last month, look for prior in last 2 years. If either of these conditions is not true, STOP. If true, take $A1C_{recent}$-$A1C_{one\ prior}$, if this is >.9 then + loss of control.
E6	**Patient is self-pay**	*…no health insurance and may have trouble affording care.*<br><br>Automated message to CCB that medical assistant contacted to direct patient to “hospital room 2b” for insurance counseling.	Order for f/u in “Insurance Coverage Clinic” placed automatically which will show to MA at time of depart process	Arrive at customized plan.	
E7	**Patient has a significant “pill burden” based on taking > 7 different medications per day**	*…been taking more than 7 different medications per day. Consider whether any can be discontinued.*	Alert with orders checked to either consult Medication Therapy Management or order a pill box. Provider selects one of these two options.	Provider and patient discuss unnecessary drugs that can reduce pill burden	

© 2022 Weiner SJ et al. *JAMA Network Open*.

Simplified rules for the CDS shown above:

E1) Missed Appointments: From T=-120 to today, check for no shows. Return the most recent 2 no-shows (date & clinic), or one, or NONE.

E4) *Loss of Control of Diabetes:* If HbA1C (lab or POCT) in last month, look for prior in last 2 years. If either of these conditions is not true, STOP. If true, take $A1C_{recent}$-$A1C_{one\ prior}$, if this is >.9 then + loss of control.

E6) *Patient is self-pay*: Is patient registered as self-pay at the current encounter?

E7) *Patient has a significant "pill burden":* Count active prescription orders that are not "product Type"="Supply". E7=this count

© 2022 Weiner SJ et al. *JAMA Network Open*.

eAppendix 5. Raw Data

Figure 1 provides raw data on the disposition of all contextual red flags and contextual factors in both trial groups, including contextual factors disclosed by patients without a clinician probe (“Factors without red flags to probe”). These disclosures occurred either because the patient reported the contextual factors on the pre-visit questionnaire or revealed them spontaneously during the encounter. In addition, red flag outcomes are shown. Red flags could be improved, worsened, mixed, or no change (a category that includes cases were evidence of change was not available). A red flag outcome could be mixed when there was more than one element -- for example, when a good outcome would require that a patient had engaged in both diet modification and exercise but at follow-up had adopted one but not the other.

Figure 1: Raw study outcomes by arm. “No change” includes cases where evidence of change was not available.

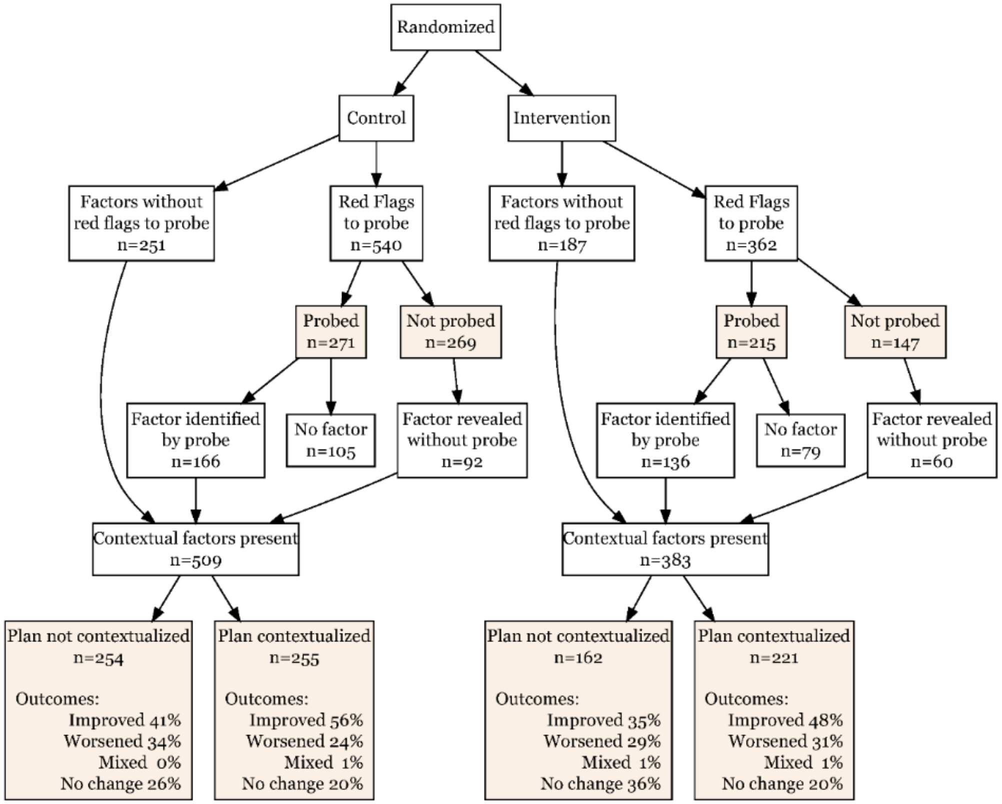

© 2022 Weiner SJ et al. *JAMA Network Open*.

eAppendix 6. Regression Tables

Regression Tables

Table 1: Multivariate regression models for main study outcomes

	**Red flag probed by provider**			**Contextual factor incorporated into care plan**			**Red flag improved at 4-6 months**			**Red flag worse at 4-6 months**		
Predictors	*Odds Ratios*	*CI*	*p*	*Odds Ratios*	*CI*	*P*	*Odds Ratios*	*CI*	*p*	*Odds Ratios*	*CI*	*p*
Intercept	0.28	0.10 – 0.80		0.24	0.05 – 1.12		1.24	0.74 – 2.11		0.51	0.32 – 0.81	
Intervention arm	2.12	1.14 – 3.93	**0.02**	2.67	1.32 – 5.41	**0.006**	0.96	0.57 – 1.63	0.88	1.31	0.84 – 2.04	0.86
Site B	4.40	2.26 – 8.58	**<0.001**	0.21	0.08 – 0.55	**0.002**	0.35	0.20 – 0.61	**<0.001**	0.77	0.48 – 1.21	0.67
Factor selected by patient pre-visit	0.15	0.07 – 0.30	**<0.001**	1.42	0.57 – 3.54	0.45						
Recorder visible	1.57	0.63 – 3.96	0.34	0.86	0.28 – 2.66	0.79						
Factor identified by probe				62	22 – 173	**<0.001**						
Factor revealed by patient				8.55	3.98 – 18.4	**<0.001**						
Factor incorporated into care plan							2.13	1.38 – 3.28	**0.001**	0.67	0.45 – 0.99	**0.046**

Note: Models include random intercepts for visit, patient, and provider.

Table 2: Multivariate regression models for effect of EMR boxes/alerts when pre-visit red flags are available, intervention arm visits

	**Red flag probed by provider**			**Contextual factor incorporated into care plan**			**Red flag improved at 4-6 months**			**Red flag worse at 4-6 months**		
Predictors	*Odds Ratios*	*CI*	*p*	*Odds Ratios*	*CI*	*p*	*Odds Ratios*	*CI*	*p*	*Odds Ratios*	*CI*	*p*
Intercept	0.13	0.04 – 0.39		1.23	0.46 – 3.29		0.55	0.21 – 1.44		1.07	0.36 – 3.20	
Site B	1.75	0.72 – 4.23	0.21	0.15	0.04 – 0.58	**0.006**	0.61	0.23 – 1.59	0.31	0.14	0.04 – 0.54	**0.004**
Box/alert	3.64	1.18 – 11.2	**0.024**	11.3	2.27 – 55.9	**0.003**	0.75	0.30 – 1.89	0.55	0.19	0.05 – 0.73	**0.016**
Factor incorporated into care plan							3.08	1.39 – 6.79	**0.005**	1.01	0.38 – 2.67	0.99

Note: Models include random intercepts for visit, patient, and provider.

© 2022 Weiner SJ et al. *JAMA Network Open*.

Table 3: Multivariate regression models for effect of EMR boxes/alerts when pre-visit red flags are available, all visits

	**Red flag probed by provider**			**Contextual factor incorporated into care plan**			**Red flag improved at 4-6 months**			**Red flag worse at 4-6 months**		
Predictors	*Odds Ratios*	*CI*	*p*	*Odds Ratios*	*CI*	*p*	*Odds Ratios*	*CI*	*p*	*Odds Ratios*	*CI*	*p*
Intercept	0.02	0.00 – 0.13		0.20	0.07 – 0.55		1.02	0.60 – 1.72		0.83	0.47 – 1.47	
Site B	2.13	1.17 – 3.88	**0.013**	0.44	0.16 – 1.18	0.10	0.45	0.27 – 0.76	**0.003**	0.35	0.19 – 0.65	**0.001**
Box/alert	17.0	2.13 – 136	**0.008**	10.8	1.89 – 61	**0.007**	0.99	0.46 – 2.16	0.98	0.31	0.11 – 0.85	**0.023**
Intervention arm	7.40	0.75 – 72.9	0.09	2.40	1.04 – 5.52	**0.040**	0.77	0.46 – 1.28	0.31	0.91	0.52 – 1.60	0.74
Intervention arm x Box/alert	0.21	0.02 – 2.19	0.19	1.54	0.15 – 16.3	0.73	0.73	0.23 – 2.26	0.58	0.92	0.23 – 3.72	0.91
Factor incorporated into care plan							2.92	1.79 – 4.75	**<0.001**	0.84	0.48 – 1.46	0.54

Note: Models include random intercepts for visit, patient, and provider.

© 2022 Weiner SJ et al. *JAMA Network Open*.

eAppendix 7. Clinician Performance in Intervention vs Control

Clinician probing, identification of contextual factors, and contextual care plan for each category of contextual red flag in control and treatment

	Total red flags				Probed (out of total)				Factors with red flags (out of total)				Plans (out of factors with red flags)			
	Control		Intervention		Control		Intervention		Control		Intervention		Control		Intervention	
Red Flag	N	%	N	%	N	%	N	%	N	%	N	%	N	%	N	%
1 – Uncontrolled chronic condition	80	15%	37	10%	49	61%	31	84%	15	19%	12	32%	9	60%	11	92%
2 – Appointment non-adherence (clinics, labs, imaging, procedures)	81	15%	59	16%	33	41%	23	39%	42	52%	26	44%	28	67%	20	77%
3 – Under/over Resource Utilization	23	4%	14	4%	6	26%	4	29%	12	52%	9	64%	7	58%	8	89%
4 - Medication non-adherence	166	31%	106	29%	74	45%	62	58%	52	31%	52	49%	39	75%	43	83%
5 – Plan of Care non-adherence	100	19%	68	19%	43	43%	38	56%	80	80%	51	75%	54	68%	38	75%
6 – Significant weight gain/loss	7	1%	4	1%	6	86%	3	75%	3	43%	2	50%	2	67%	2	100%
7 - Lack of knowledge of health or health care status	3	1%	6	2%	3	100%	5	83%	3	100%	3	50%	3	100%	2	67%
8 - Medical equipment/supplies non-adherence	19	4%	19	5%	13	68%	12	63%	15	79%	15	79%	11	73%	10	67%
9 - Other	61	11%	49	14%	44	72%	37	76%	36	59%	26	53%	27	75%	22	85%
Total	540	100%	362	100%	271	50%	215	59%	258	48%	196	54%	180	70%	156	80%

© 2022 Weiner SJ et al. *JAMA Network Open*.

eAppendix 8. Contextual Red Flags, "Other" Category

Contextual Red Flags, "Other" Category:

1. Patient states, "I am stressing man with these bills, and I don't have my wife to move me up."
2. Patient states, "I went crazy for the fourth and spent a lot of money. I was breaking out of the stage of the other stuff and now I am suffering from that."
3. Patient states, "I only eat one meal a day because I don't have time."
4. Patient states, "I don't feel okay mentally. I cry excessively and feel depressed."'
5. Patient states, "I am not depressed, but there are days that I am unhappy, and I get overwhelmed."
6. Patient states, "There is no memory left anymore. I can't have a conversation because words just slip my mind."
7. Patient states, "My husband and I don't conversate anymore. I am just there to do wifely duties. Does it bother me? To a degree."'
8. Patient states, "If it's not one thing it's another so what's the reason of me even being here."'
9. Patient doesn't want to respond to certain questions about their mood.
10. Patient sighs heavily, says, "I've been going through some stuff."
11. Patient mentioned that are dealing with stress at work.
12. Patient comments they are worried about having cancer.
13. Patient comments, "My job is stressful and demanding and the winter makes me sad."
14. Patient signs and says, "I've been better..."
15. Patient is indicating they want to delay medical care/procedures.
16. Patient comments they did not get IUD removed 3 years ago when they were supposed to.
17. Patient comments, "It's been a weird couple of years..."
18. Patient comments that she has worsening depression.
19. Patient mentions they have been unable to use ergonomic chair at work
20. Patient comments that they suffer from depression
21. Patient mentions that stress is impacting ability to manage health
22. Patient comments that they have grieving from many close people passing
23. Patient comments they are having issues with their sister
24. With no context, patient says they want a blood test for Lyme disease (i.e., request comes out of the blue
25. Patient mentions that she is having trouble with her bipolar daughter.
26. Patient mentions that her sister being illness is affecting her (patient's) health.
27. Patient comments that they are on lots of meds.
28. Patient discloses that her son was killed, and she is dealing with court issues.
29. Patient mentions she has been unable to get mental health care.
30. Patient taking care of an ill son with bipolar disease.
31. Patient got notice that she needed a colonoscopy but doesn't think she does.
32. Patient hesitant to come back for recommended blood pressure check.
33. Patient's wife passed.
34. Patient lost parents recently.
35. Patient has had "many deaths" in her family.
36. Patient mentions "emotional challenges."
37. Patient says, "…a lot's going on with my family."
38. Patient didn't follow up with neurology as previously discussed.

© 2022 Weiner SJ et al. *JAMA Network Open*.

39. Patient comments, “I've been a bad girl...”
40. Patient comment’s that his wife lost her job and insurance.
41. Patient comments that she's stressed.
42. Patient comments that he hasn’t tried the nicotine gum that was prescribed previously.
43. Patient says about himself, “I'm disappointed.”
44. Patient comments that they are under stress – “having a tough time.”
45. Patient comments they are not drinking enough water.
46. Patient comments they are under a lot of stress.
47. Patient says that “there is a lot going on.”
48. Patient starts sobbing near end of visit.
49. Patient comments that having a young child affects sleep and opportunities to exercise.
50. Patient comments that looking for a job is causing stress.
51. Patient says that they would like to be progressing more than he is but does not want people to 'lecture' him about it all the time.
52. Patient starts sobbing during the encounter.
53. Patient who is still in emotional distress says she wants to wean herself off of counseling and mental health meds.
54. Patient mentions that she is having emotional issues following a divorce.
55. Patient mentions several times that his memory is bad.
56. Patient discloses that he did not tell that doctor about hemorrhoids that were already an issue at the previous visit.
57. Patient reports that she resigned from her job because it was stressful and dangerous.
58. Patient mentions that she is out of work.
59. Patient who is taking care of ill husband comments that she is stressed.
60. Patient says she wants to skip getting ordered lab work.
61. Patient mentions they are in a difficult living situation.
62. Patient comments that they are having emotional issues, including anxiety.
63. Patient says they are having family issues at home.
64. Patient reports they are experiencing stress trying to manage their health.
65. Patient says they are having trouble with transportation
66. Patient reports experiencing stress at work.
67. Patient has stress about condition/worsening symptoms.
68. Patient comments that anxiety is affecting her life.
69. Patient says they stopped the home health care aid services they’d been receiving.
70. Patient said they felt like they were having a panic attack and got emotional.
71. Patient has appointments scheduled that she isn't aware of.
72. Patient mentioned she’d had a joint replacement that was affecting her.
73. Patient says, “My husband has a new diagnosis of atrial fibrillation and there is a lot of uncertainty about his heart health.”
74. Patient says that “acting as the primary caretaker for my 93-year-old mother is causing me stress and time pressures.”
75. Patient comments that recently they’ve been having difficulty sleeping.
76. Patient says: “At the present time I am dealing with my son who was sent home on hospice. Taking care of him is so overwhelming that I forget to take care of myself sometimes. The nursing home I tried to put him in denied him. I am now getting ready to start with another nursing home. With all my sick issues: diabetes, hypertension, asthma, thyroid it is so overwhelming.”
77. Patient says, “I’m a little overwhelmed with helping my mother who is sick and needs help. Sometimes I'm a little depressed and just can’t cope.”

© 2022 Weiner SJ et al. *JAMA Network Open*.

78. Patient comments, “My husband has so many wound problems. And recently I was diagnosed with stage 1 breast cancer and that's scary too.”
79. Patient comments that they are moving and perhaps retiring.
80. Patient comments, “I am always taking care of others.”
81. Patient declined pneumonia vaccine said they’d also decline and any other vaccine.
82. Patient comments that they are worried sometimes that they have things to do but don't have the money to do it.
83. Patient comments that they are about to run out of meds after changing insurance.
84. Patient reveals they are in jeopardy of being homeless.
85. Patient comments without clear reason that there is a nun in her community who has Parkinson's and is choosing to ignore it.
86. Patient says she is depressed and doesn't trust the surgeon who is supposed to do her hysterectomy.
87. Patient comments that her daughter revealed that she was molested by a sitter.
88. Patient says he's overwhelmed – and then almost cries.
89. Patient comments that she is depressed.
90. Patient asking for ibuprofen refill even though other doctor declined to refill prescription because of concerns about adverse effects.
91. Patient says she is stressed from taking care of her mother. Then says “I have that anxiety....”
92. Patient reveals she’s taking care of an ill husband.
93. Patient comments that she doesn’t want to start treating her condition for a couple of months.
94. Patient states they are having family hardships, then says “it's been rough” and cries.
95. Caregiver, also at visit, says she is overwhelmed, and that patient won't ask for help.
96. Patient says ”Uh oh, I'm guilty!” as provider enters.
97. Patient talks about being upset/grieving because he has lost of family members due to Covid.
98. Patient comments that her home situation is stressful
99. Patient says she doesn't believe she has cancer despite diagnosis.
100. Patient says he has recently started taking care of a family member.
101. Patient says he is having difficulty managing his health, especially his weight
102. Patient comments he has very big medical bills.
103. Patient share that he is going through a divorce.
104. Patient mentions that he is taking care of a disabled brother.
105. Patient says she lost weight recently because she was stressed out because of this of appointment
106. Patient discloses that they have anxiety, stress, and depression.

© 2022 Weiner SJ et al. *JAMA Network Open*.

eAppendix 9. Path Analytic Summary of Multivariate Models

Figure 1: Path analytic summary of multivariate models. Each node with arrows pointing to it represents a separate mixed effects model in which the node's outcome is regressed on the nodes pointing to it in the diagram. Illustrates the direct and indirect effects of the intervention on contextual probing, identification of contextual factors, contextualization of care plans, and patient outcomes.

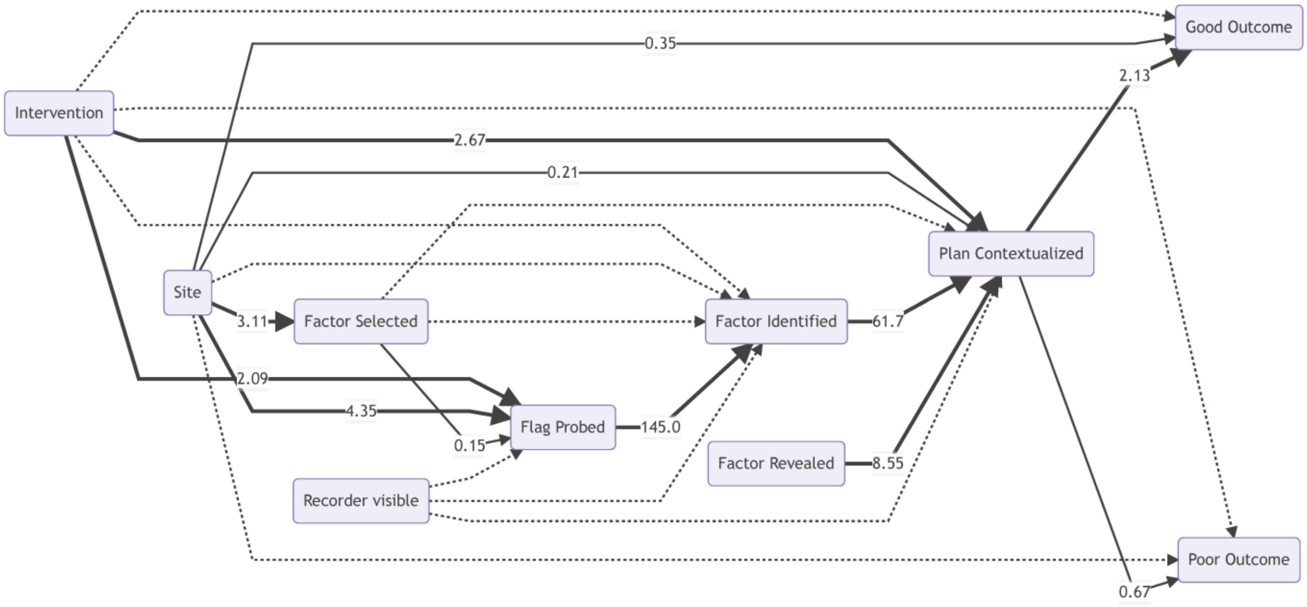

© 2022 Weiner SJ et al. *JAMA Network Open*.
