# Supplementary material for: Effect of Electronic Health Record Clinical Decision Support on Contextualization of Care: A Randomized Clinical Trial
Source: JAMA Netw Open. 2022 Oct 24;5(10):e2238231. doi: 10.1001/jamanetworkopen.2022.38231 (PMC9593230; doi:10.1001/jamanetworkopen.2022.38231)
Supplement: Supplement 3. — Data Sharing Statement [file jamanetwopen-e2238231-s003.pdf]

Data Sharing Statement

Weiner. Effect of Electronic Health Record Clinical Decision Support on Contextualization of Care. *JAMA Netw Open*. Published October 24, 2022.
doi:10.1001/jamanetworkopen.2022.38231

Data
Data available: Yes
Data types: Deidentified participant data
How to access data: Send requests to sweiner@uic.edu
When available: With publication

Supporting Documents
Document types: Statistical/analytic code
How to access documents: Send request to sweiner@uic.edu
When available: With publication

Additional Information
Who can access the data: researchers whose proposed use of the data has been approved
Types of analyses: for research purposes
Mechanisms of data availability: With investigator support
